# Modeling of Environmental Effects in Genome-Wide Association Studies Identifies *SLC2A2* and *HP* as Novel Loci Influencing Serum Cholesterol Levels

**DOI:** 10.1371/journal.pgen.1000798

**Published:** 2010-01-08

**Authors:** Wilmar Igl, Åsa Johansson, James F. Wilson, Sarah H. Wild, Ozren Polašek, Caroline Hayward, Veronique Vitart, Nicholas Hastie, Pavao Rudan, Carsten Gnewuch, Gerd Schmitz, Thomas Meitinger, Peter P. Pramstaller, Andrew A. Hicks, Ben A. Oostra, Cornelia M. van Duijn, Igor Rudan, Alan Wright, Harry Campbell, Ulf Gyllensten, EUROSPAN Consortium

**Affiliations:** 1 Department of Genetics and Pathology, Rudbeck Laboratory, University of Uppsala, Uppsala, Sweden; 2 Centre for Population Health Sciences and Institute of Genetics and Molecular Medicine, University of Edinburgh, Edinburgh, United Kingdom; 3 Andrija Štampar School of Public Health, Faculty of Medicine, University of Zagreb, Zagreb, Croatia; 4 MRC Human Genetics Unit, Institute of Genetics and Molecular Medicine, University of Edinburgh, Edinburgh, United Kingdom; 5 Institute for Anthropological Research, Zagreb, Croatia; 6 Institute for Clinical Chemistry and Laboratory Medicine, Regensburg University Medical Center, Regensburg, Germany; 7 Helmholtz Zentrum München, GmbH, Neuherberg, Munich, Germany; 8 Institute of Genetic Medicine, European Academy Bozen/Bolzano (EURAC), Bolzano, Italy; 9 Institute of Genetic Medicine, University of Lübeck, Lübeck, Germany; 10 Department of Neurology, General Central Hospital, Bolzano, Italy; 11 Department of Neurology, University of Lübeck, Lübeck, Germany; 12 Department of Clinical Genetics, Erasmus University Medical Center, Rotterdam, The Netherlands; 13 Department of Epidemiology, Erasmus University Medical Center, Rotterdam, The Netherlands; 14 Croatian Centre for Global Health, Faculty of Medicine, University of Split, Split, Croatia; IRCCS Burlo Garofolo, University of Trieste, Italy

## Abstract

Genome-wide association studies (GWAS) have identified 38 larger genetic regions affecting classical blood lipid levels without adjusting for important environmental influences. We modeled diet and physical activity in a GWAS in order to identify novel loci affecting total cholesterol, LDL cholesterol, HDL cholesterol, and triglyceride levels. The Swedish (SE) EUROSPAN cohort (*N*_SE_ = 656) was screened for candidate genes and the non-Swedish (NS) EUROSPAN cohorts (*N*_NS_ = 3,282) were used for replication. In total, 3 SNPs were associated in the Swedish sample and were replicated in the non-Swedish cohorts. While SNP rs1532624 was a replication of the previously published association between CETP and HDL cholesterol, the other two were novel findings. For the latter SNPs, the *p-*value for association was substantially improved by inclusion of environmental covariates: SNP rs5400 (*p*_SE,unadjusted_ = 3.6×10^−5^, *p*_SE,adjusted_ = 2.2×10^−6^, *p*_NS,unadjusted_ = 0.047) in the *SLC2A2* (Glucose transporter type 2) and rs2000999 (*p*_SE,unadjusted_ = 1.1×10^−3^, *p*_SE,adjusted_ = 3.8×10^−4^, *p*_NS,unadjusted_ = 0.035) in the *HP* gene (Haptoglobin-related protein precursor). Both showed evidence of association with total cholesterol. These results demonstrate that inclusion of important environmental factors in the analysis model can reveal new genetic susceptibility loci.

## Author Summary

In this article we report a genome-wide association study on cholesterol levels in the human blood. We used a Swedish cohort to select genetic polymorphisms that showed the strongest association with cholesterol levels adjusted for diet and physical activity. We replicated several genetic loci in other European cohorts. This approach extends present genome-wide association studies on lipid levels, which did not take these lifestyle factors into account, to improve statistical results and discover novel genes. In our analysis, we could identify two genetic loci in the S*LC2A2* (Glucose transporter type 2) and the *HP* (Haptoglobin-related protein precursor) gene whose effects on total cholesterol have not been reported yet. The results show that inclusion of important environmental factors in the analysis model can reveal new insights into genetic determinants of clinical parameters relevant for metabolic and cardiovascular disease.

## Introduction

Genome-wide association studies (GWAS) have identified more than 38 larger genetic regions which influence blood levels of total cholesterol (TC), low-density lipoprotein cholesterol (LDL-C), high-density lipoprotein cholesterol (HDL-C) and triglycerides (TG) [1]–[3]. These studies modeled basic anthropometric confounders, such as sex and age, while leaving out important environmental influences, such as diet and activity. This strategy is statistically suboptimal since the unexplained variation in the phenotype can increase the measurement error and as a result require larger sample sizes to detect a significant effect. Manolio [4] argued strongly for modeling of environmental covariates in GWAS and recommended lipid levels as a paradigmatic phenotype for studying the genetic and environmental architecture of quantitative traits.

In order to explore the usefulness of including both environmental and genetic factors in the analysis model, we used lipid measurements from the EUROSPAN study, comprising 3,938 individuals for whom genome-wide SNP data (*N*_SNP_ = 311,388) were available [5]. We measured daily intake of food and physical activity at work and at leisure and modeled the influence of those environmental covariates on serum lipid levels in a GWAS. First, data from the Northern Sweden Population Health Study (NSPHS) were used as a discovery cohort to screen for SNPs that displayed the lowest *p*-values when the model was adjusted for environmental covariates. We then used the other, non-Swedish EUROSPAN cohorts for replication of our strongest associations in a candidate gene association study (CGAS).

We chose a population living in northern Sweden for the selection of candidate loci because it shows strong natural heterogeneity in certain lifestyle factors (e.g. diet, activity), but homogeneity in other environmental aspects such as climate [6]. Whereas one group is living a modern, sedentary lifestyle found also in the southern part of Sweden and other western European countries, a subgroup of Swedes follows a traditional, semi-nomadic way of life based on reindeer herding. Reindeer herders typically show higher intake of game meat (reindeer, moose), which has a high protein and low fat content, and lower intake of non-game meat, fish, and dairy products among other, lesser differences. They also exert more physical activity at work to tend their reindeer herds, but less activity at leisure [7].

## Results

### Exploratory GWAS in NSPHS

We performed a GWAS with a lifestyle-adjusted model which included not only sex and age, but also daily intake of game meat, non-game meat, fish, milk products, physical activity at work and at leisure as covariates. We focused on the 0.05% of all SNPs with the lowest *p*-values in the diet- and activity-adjusted model (corresponding to about 150 SNPs per lipid). For total cholesterol, 88 of these were located in a gene and 14 in genes that have been associated with energy metabolism (http://www.ncbi.nlm.nih.gov/omim/). For LDL-C, 65 SNPs were located in a gene, of which 8 were functionally relevant. Several of the SNPs for LDL-C were identical with those affecting total cholesterol, as expected from the high correlation (*r* = 0.91) between both phenotypes. For HDL-C, SNP rs2292883, located in the *MLPH* gene (Melanophilin), showed a genome-wide significant *p*-value (*p* = 1.06×10^−07^). 69 SNPs for HDL-C were located in a gene and 14 of those genes were reported as having a metabolic effect. Finally, for triglycerides, 63 SNPs were located in a gene, but only 4 SNPs in genes with a functional annotation of interest (Table 1 and Table S1A, S1B, S1C, S1D).

**Table 1:** Candidate SNPs (*n* = 39) selected from the Swedish discovery cohort.

SNP	*p*-value, unadjusted^a^	*p*-value, adjusted^b^	*p*-value ratio^c^	Gene Symbol	Product name (Product Symbol)
**TC**					
**rs10513684**	**2.91E-05**	**1.08E-06**	**27**	**SLC2A2**	**Glucose transporter type 2 (GLUT-2)**
rs1684885	3.47E-03	2.01E-04	17	PRKCI	Protein kinase C iota type (nPKC-iota)
***rs5400***	***3.57E-05***	***2.18E-06***	***16***	***SLC2A2***	***Glucose transporter type 2 (GLUT-2)***
rs47137	3.69E-03	2.63E-04	14	SLC2A12	Glucose transporter type 12 (GLUT-12)
rs669552	3.46E-03	2.87E-04	12	FNDC3B	Factor for Adipocyte Differentiation 104
rs2303324	1.49E-03	1.65E-04	9	GALNT14	Polypeptide GalNAc transferase 14
rs12617790	2.26E-03	2.53E-04	9	GALNT14	Polypeptide GalNAc transferase 14
rs10041333	3.04E-03	3.74E-04	8	FABP6	Gastrotropin (GT), alt. Fatty Acid-Binding Protein 6
rs222014	2.93E-03	3.71E-04	8	GC	Vitamin D-binding protein Precursor (DBP)
***rs2000999***	***1.12E-03***	***3.84E-04***	***3***	***HP***	***Haptoglobin-related protein Precursor***
rs2070657	1.70E-04	1.43E-04	1	APP	Alzheimer disease amyloid protein (ABPP)
rs2186830	2.66E-04	2.78E-04	1	COLEC12	Collectin-12
rs2478571	3.07E-04	4.42E-04	1	SLC39A12	Zinc transporter (ZIP12)
**LDL-C**					
rs1684885	1.53E-04	1.61E-05	10	PRKCI	Protein kinase C iota type (nPKC-iota)
rs1684881	2.80E-04	3.14E-05	9	PRKCI	Protein kinase C iota type (nPKC-iota)
**rs10513684**	**1.98E-04**	**2.61E-05**	**8**	**SLC2A2**	**Glucose transporter type 2 (GLUT-2)**
**rs5400**	**4.22E-04**	**6.10E-05**	**7**	**SLC2A2**	**Glucose transporter type 2(GLUT-2)**
rs12617790	1.65E-03	2.96E-04	6	GALNT14	Polypeptide GalNAc transferase 14
rs7583934	2.78E-04	1.84E-04	2	LRP1B	Low-density lipoprotein receptor-related protein (LRP-DIT)
rs1864616	8.64E-05	1.36E-04	2	TGFBR2	Transforming growth factor-beta receptor type II (TGFR-2)
rs843319	6.74E-05	2.89E-04	4	MBOAT1	O-acyltransferase domain-containing protein 1
**HDL-C**					
rs2292883	1.48E-06	1.06E-07*	14	MLPH	Melanophilin
rs12712846	1.14E-03	3.02E-04	4	MTA3	Metastasis-associated protein
rs365578	8.39E-04	2.92E-04	3	NDUFS4	NADH dehydrogenase 8 iron-sulfur protein 4 (CI-AQDQ)
**rs9866473**	**4.34E-04**	**2.55E-04**	**2**	**CETP**	**Cholesteryl ester transfer protein**
rs10519336	6.34E-04	3.82E-04	2	MCC	Colorectal mutant cancer protein
rs2054247	3.73E-05	3.70E-05	1	APLP2	Amyloid-like protein 2 Precursor
rs11708205	2.67E-04	3.57E-04	1	PLD1	Phospholipase D1
**rs9863761**	**5.20E-06**	**7.74E-06**	**1**	**CETP**	**Cholesteryl ester transfer protein**
**rs2124147**	**1.49E-04**	**2.23E-04**	**2**	**CETP**	**Cholesteryl ester transfer protein**
rs1567385	1.32E-04	1.99E-04	2	MAP4K4	Mitogen-activated protein kinase 4 (MEKKK4)
rs3776817	6.82E-05	1.31E-04	2	ADAMTS2	Procollagen I N-proteinase
rs1999088	7.19E-05	1.51E-04	2	MBNL2	Muscleblind-like protein 2
rs1782644	1.39E-04	3.10E-04	2	ZMIZ1	Zinc finger MIZ domain-containing protein 1
***rs1532624***	***1.06E-06***	***2.55E-06***	***2***	***CETP***	***Cholesteryl ester transfer protein***
**TG**					
rs4304239	1.63E-03	2.40E-04	7	IGF2BP3	Insulin-like growth factor 2 mRNA-binding protein 3
rs11770192	1.82E-03	2.40E-04	8	IGF2BP3	Insulin-like growth factor 2 mRNA-binding protein 3
rs12540730	7.79E-04	2.43E-04	3	IGF2BP3	Insulin-like growth factor 2 mRNA-binding protein 3
rs3823763	9.58E-05	4.45E-05	2	BBS9	Parathyroid hormone-responsive B1 gene protein (PTHB1)

All candidate SNPs show strongest associations (*p*-value, top 0.05% SNPs per lipid trait) and are located in a gene which has been reported to be relevant for energy metabolism. SNPs are sorted by *p*-value ratio (unadjusted∶unadjusted).

*: *p*≤1.6E-07 = genome-wide significant; All SNPs in genes with at least one replicated SNP are displayed in bold, replicated SNPs are formated in bold italics. a) unadjusted = covariates include sex and age; b) adjusted = covariates include sex, age, game meat, non-game meat, fish, milk products, physical activity at work and at leisure; c) *p*-ratio = max(*p*_unadjusted_∶*p*_adjusted_; *p*_adjusted_∶*p*_unadjusted_); *p*_unadjusted_∶*p*_adjusted_ ratios are aligned left, *p*_adjusted_∶*p*_unadjusted_ ratios are aligned right.

### *P*-value changes

**Figure 1:**
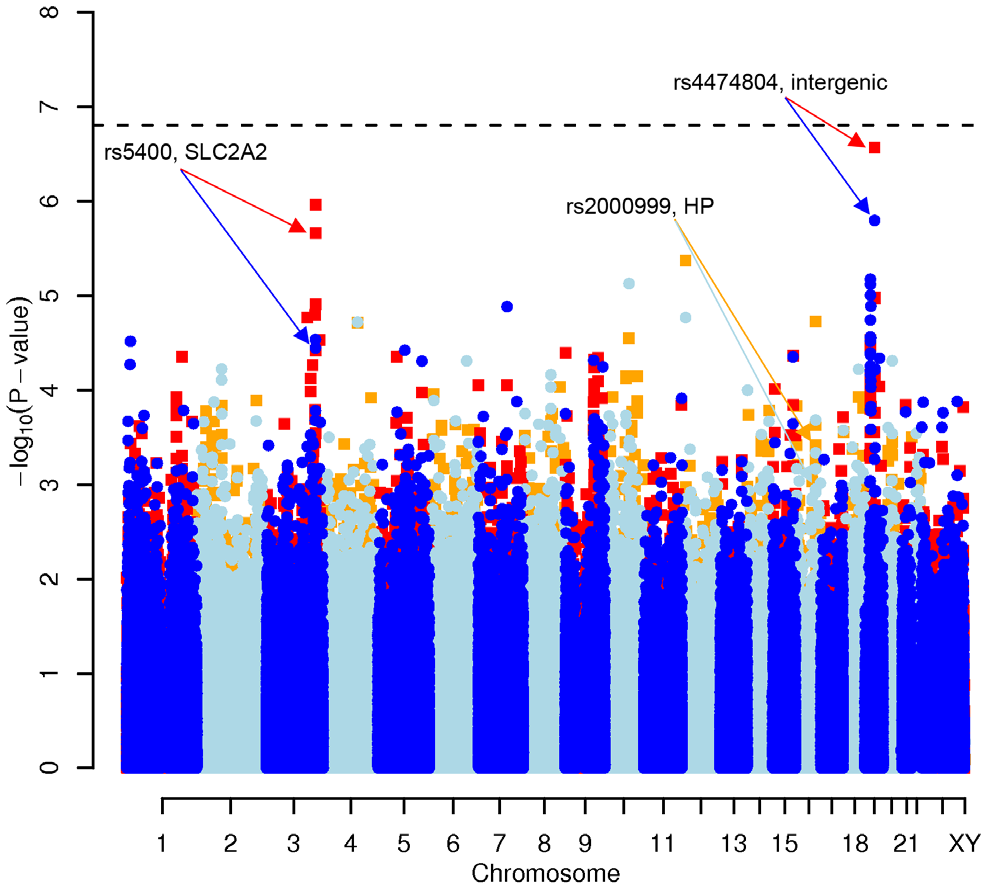
Manhattan plot of genome-wide effects on total cholesterol levels in the Swedish discovery cohort. Results for two GWAS analysis models are presented. The unadjusted model (dark blue and light blue circles) included only sex and age as covariates. The adjusted model (red and orange squares) additionally contained food intake and physical activity as predictors. The dashed line indicates the local Bonferroni-adjusted α error = 1.6×10^−7^.

**Figure 2:**
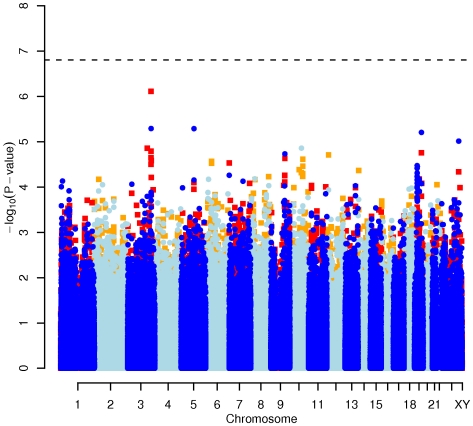
Manhattan plot of genome-wide effects on LDL cholesterol levels in the Swedish discovery cohort. Results for two GWAS analysis models are presented. The unadjusted model (dark blue and light blue circles) included only sex and age as covariates. The adjusted model (red and orange squares) additionally contained food intake and physical activity as predictors. The dashed line indicates the local Bonferroni-adjusted α error = 1.6×10^−7^.

**Figure 3:**
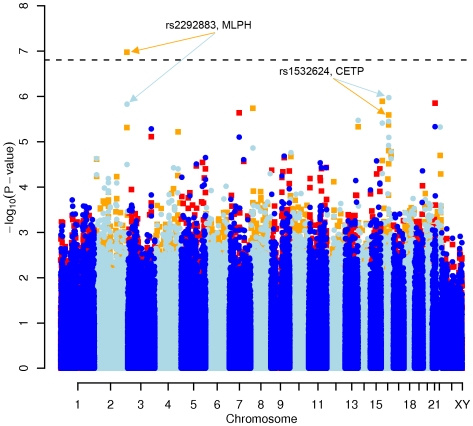
Manhattan plot of genome-wide effects on HDL cholesterol levels in the Swedish discovery cohort. Results for two GWAS analysis models are presented. The unadjusted model (dark blue and light blue circles) included only sex and age as covariates. The adjusted model (red and orange squares) additionally contained food intake and physical activity as predictors. The dashed line indicates the local Bonferroni-adjusted α error = 1.6×10^−7^.

**Figure 4:**
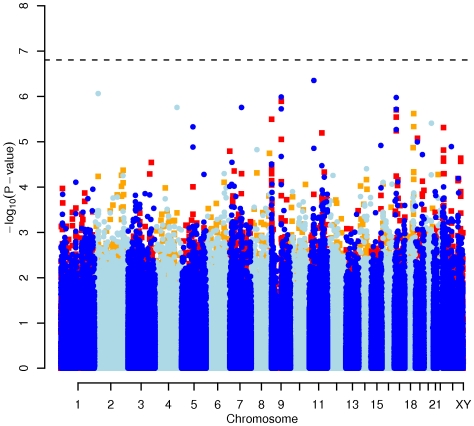
Manhattan plot of genome-wide effects on triglyceride levels in the Swedish discovery cohort. Results for two GWAS analysis models are presented. The unadjusted model (dark blue and light blue circles) included only sex and age as covariates. The adjusted model (red and orange squares) additionally contained food intake and physical activity as predictors. The dashed line indicates the local Bonferroni-adjusted α error = 1.6×10^−7^.

In order to evaluate the effect of including diet and activity covariates in the association analysis, we overlaid the *p-*values in the Manhattan plots from the NSPHS for the unadjusted and adjusted GWAS models (Figure 1, Figure 2, Figure 3, Figure 4). More refined GWAS results separating the effect of adjusting for either diet or physical activity are presented in Figure S1A, S1B, S1C, S1D; and Figure S2A, S2B, S2C, S2D. As expected, the *p-*values for a number of SNPs were sensitive to the inclusion of both diet and activity covariates in the model. We matched the 0.05% SNPs with the lowest *p*-values (top SNP list) between the unadjusted and the adjusted model. For TC, 83 (53%) SNPs were found in both top SNP lists. Those lists contained 102 (64%) identical SNPs for LDL-C and 103 (65%) for HDL-C. The analyses resulted in the same 74 (47%) top SNPs for TG levels (Table S1A, S1B, S1C, S1D). Finally, we compared the *p*-value changes of the resulting 39 candidate SNPs that are located in genes with a metabolic effect between the diet and activity-adjusted (full) model and the unadjusted (restricted) model resulting in an up to 27-fold *p*-value decrease (Table 1).

### Confirmatory CGAS in EUROSPAN

A food- and activity-adjusted candidate gene association study of the final 39 candidate SNPs in the Scottish (SC) sample (*N* = 714) was applied using similar lifestyle covariates (Table 2; Table S1E, S1F, S1G, S1H; Table S2). We replicated the effect of rs2000999 (*p*_SC,unadj_ = 6.16×10^−03^, *p*_SC,adj_ = 4.33×10^−03^) in the *HP* gene (Haptoglobin-related protein Precursor) on TC level and the effect of rs1532624 (*p*_SC,unadj_ = 2.40×10^−09^, *p*_SC,adj_ = 1.96×10^−09^) in *CETP* (Cholesteryl ester transfer protein) on HDL-C. In the Swedish cohort (SE), the unadjusted genetic effect of rs2000999 in the *HP* gene is equivalent to a moderately large difference in average TC level of 20.21 mg/dl between the homozyguous genotypes (Mean_SE,unadj_(TC|A/A)−Mean_SE,unadj_(TC|G/G) = 243.16−222.95, Effect Size_SE,unadj_ = 0.41, Effect Size_SE,adj_ = 0.44)(Effect Size (*ES*) = (*M*_A/A_−*M_B/B_*)/*SD*_pooled_). Equivalent effects were observed in the Scottish replication sample (*M*_SC,unadj_(TC|A/A)−*M*_SC,unadj_(TC|G/G) = 235.36 mg/dl−222.54 mg/dl = 12.82 mg/dl, *ES*_SC,unadj_ = 0.29, *ES*_SC,adj_ = 0.52). SNP rs1532624 in the *CETP* gene is associated with a large, unadjusted difference in HDL-C level of 9.99 mg/dl (*M*_SE,unadj_(HDL-C|A/A)−*M*_SE,unadj_(HDL-C|C/C) = 68.14 mg/dl−58.15 mg/dl, *ES*_SE,unadj_ = 0.73, *ES*_SE,adj_ = 0.48) in the discovery cohort and similar effects regarding direction and size in the replication cohort (*M*_SC,unadj_(HDL-C|A/A)−*M*_SC,unadj_(HDL-C|C/C) = 69.79 mg/dl−60.75 mg/dl = 9.04 mg/dl; *ES*_SC, unadj_ = 0.59, *ES*_SC, adj_ = 0.57).

**Table 2:** SNPs (*n* = 3) discovered in a Swedish and replicated in a non-Swedish EUROSPAN cohort.

SNP	Gene	Trait	Cohort	*p*-value, unadjusted^a^	*p*-value, adjusted^b^	Mean Difference, unadjusted^c^	Effect Size, unadjusted^d^	Effect Size, adjusted^e^
rs2000999	HP	TC	Discovery, SE	1.12E-03	3.84E-04	20.21 mg/dl	0.41	0.44
			Replication, SC	6.16E-03	4.33E-03	12.82 mg/dl	0.29	0.52
rs1532624	CETP	HDL-C	Discovery, SE	1.06E-06	2.55E-06	9.99 mg/dl	0.73	0.48
			Replication, SC	2.40E-09	1.96E-09	9.04 mg/dl	0.59	0.57
rs5400	SLC2A2	TC	Discovery, SE	3.57E-05	2.18E-06	27.11 mg/dl	0.57	0.66
			Replication, NS	4.68E-02	N.A.	13.35 mg/dl	0.30	N.A.

For all replicated SNPs *p*-values, mean differences, and effects sizes for unadjusted and adjusted lipid levels between homozygous genotypes are reported except for replication cohort NS. Discovery Cohort SE: Swedish EUROSPAN cohort (*N*_SE_ = 656), Replication Cohort SC: Scottish EUROSPAN cohort (*N*_Sc_ = 714), Replication Cohort NS: Non-Swedish EUROSPAN cohorts (Scotland, Croatia, Italy, Netherlands, *N*_NS_ = 3,282), N.A.: not available; a) unadjusted: covariates include sex and age, b) adjusted: covariates include sex, age, game meat, non-game meat, fish, milk products, physical activity at work and at leisure, c) Genetic effect as mean difference of unadjusted lipid levels between homozygous genotypes: *M*(A/A)−*M*(B/B), d) Genetic effect as standardized effect size of unadjusted lipid levels: *ES* = (*M*_A/A_−*M*_B/B_)/*SD*_pooled_, e) Genetic effect as standardized effect size of adjusted lipid levels: *ES* = (*M*_A/A_−*M*_B/B_)/*SD*_pooled_.

We also performed an unadjusted candidate gene analysis of the 39 candidate SNPs in all non-Swedish (NS) EUROSPAN cohorts (Scotland, Croatia, The Netherlands, and Italy, *N*_NS_ = 3,282) and aggregated the results in a meta-analysis (Table 2; Table S1I, S1J, S1K, S1L). We confirmed the effects of rs5400 (*p_NS_* = 4.68×10^−02^) in *SLC2A2* on TC. We again found that rs2000999 (*p*_NS,unadj_ = 3.54×10^−2^) in *HP* influences TC levels and rs1532624 (*p*_NS,unadj_ = 2.87×10^−20^) in *CETP* (Cholesteryl ester transfer protein) affects HDL-C levels. The unadjusted genetic effect of rs5400 is equivalent to a moderately large difference in mean TC level of 27.11 mg/dl between homozyguous genotypes (*M*_SE,unadj_(TC|A/A)−*M*_SE,unadj_(TC|G/G) = 249.30 mg/dl−222.19 mg/dl, *ES*_SE,unadj_ = 0.57, *ES*_SE,adj_ = 0.66) in the Swedish Cohort and a small total effect in all non-Swedish samples (*M*_NS,unadj_(TC|A/A)−*M*_NS,unadj_(TC|G/G) = 236.69 mg/dl−223.34 mg/dl = 13.35 mg/dl, *ES*_NS,unadj_ = 0.30).

No other associations, including LDL cholesterol or triglycerides levels, were replicated (all *p*>0.05). The genome-wide significant SNP rs2292883 in the Melanophilin (*MLPH*) gene found in the Swedish cohort was not confirmed.

## Discussion

Environmental covariates may either act as moderators, mediators or even suppressors, thereby affecting the discovery of genetic susceptibility loci [8],[9]. Therefore, we conducted a GWAS, modeling genetic and important environmental effects, such as food intake and physical activity, on serum levels of classical lipids. To our knowledge, this is the first GWAS on blood lipid levels modeling environmental factors, in particular major food categories and physical activity, in international cohorts. Our analysis replicated one known locus in the *CETP* gene [1] and identified two other gene loci in the *SLC2A2* and *HP* gene, respectively, involved in energy metabolism but not previously reported to be associated with cholesterol levels.

*SLC2A2* encodes the facilitated glucose transporter member 2 (GLUT-2, Solute carrier family 2) and is predominantly expressed in the liver. Mice deficient in GLUT-2 are hyperglycemic and have elevated plasma levels of glucagon and free fatty acids [10]. Mutations in GLUT-2 cause the Fanconi-Bickel syndrome (FBS) characterized by hypercholesterolemia and hyperlipidemia [11],[12]. Cerf [13] argued that a high-fat diet causes a decreased expression of the GLUT-2 glucose receptor on β-cell islets. As a result, glucose stimulation of insulin exocytosis is impaired causing hyperglycemia, a clinical hallmark of type 2 diabetes. In addition, Kilpelainen et al. [14] found that physical activity moderates the genetic effect of *SLC2A2* on type 2 diabetes. These studies suggest that these lifestyle factors could have masked genetic effects in previous, unadjusted GWAS. This is emphasized by the strong increase in statistical significance of the *SLC2A2* polymorphisms after adjusting for diet and physical activity, indicating that the examined lifestyle factors modified the effect of this gene. Our supplemental results show that physical activity markedly moderated the genetic effect on total cholesterol.

The *HP* gene encodes the Haptoglobin-related Protein Precursor (Hp), which binds hemoglobin (Hb) to form a stable Hp-Hb complex and, thereby, prevents Hb-induced oxidative tissue damage. Asleh et al. [15] identified severe impairment in the ability of Hp to prevent oxidation caused by glycosylated Hb. Diabetes is also associated with an increase in the non-enzymatic glycosylation of serum proteins, so these authors suggested that there is a specific interaction between diabetes, cardiovascular disease and the Hp genotype. It results from the increased need of rapidly clearing glycosylated Hb-Hp complexes from the subendothelial space before they oxidatively modify low-density lipoprotein to form the atherogenic oxidized low-density lipoprotein. The *p-*value for association between the *HP* SNP rs2000999 and total serum cholesterol concentration decreased in the model adjusted for diet and physical activity, suggesting that the genetic effect is moderated by diet and physical activity. Our supporting material points out the moderating role of physical activity in particular.

We also observed a highly significant association between rs1532624 in *CETP* and HDL-C levels. The *CETP* protein catalyzes the transfer of insoluble cholesteryl esters among lipoprotein particles. Variation in *CETP* is known to affect the susceptibility to atherosclerosis and other cardiovascular diseases [16]. Adjustment for diet and physical activity in our model caused an increase of the *p*-value of this SNP. Our supporting results indicate that the genetic effect is mediated by diet or by physical activity in a similar way.

This study also has some limitations. First, we are aware that our candidate gene association approach covers only a very small fraction of all genomic loci, which is one of the potential reasons why some classical lipid-influencing genes, such as *APOE*, are not represented in our candidate SNP list. Therefore, our approach is not comprehensive and may have failed to identify other relevant lifestyle-sensitive genetic variants. Nonetheless, we decided to apply this approach to make the best out of the available lifestyle data. Second, our study provides only limited information on the role of individual lifestyle factors for a genetic variant. However, in this study we aimed at amplifying genetic effects by adjusting for a maximum amount of environmental variance in a single model and, therefore, we neglected some of these aspects here. Third, we did not model genetic covariates in known lipid-relevant genes which may also moderate the effect of other genetic predictors. This is due to the focus of this paper on gene-environment relationships.

In summary, we have demonstrated that modeling environmental factors, in particular major food categories and physical activity, can improve statistical power and lead to the discovery of novel susceptibility loci. Such models also provide an understanding of the complex interplay of genetic and environmental factors affecting human quantitative traits. Inclusion of environmental covariates represents a much needed next step in the quest to model the complete environmental and genetic architecture of complex traits.

## Methods

### Ethics statement

All EUROSPAN studies were approved by the appropriate research ethics committees according to the Declaration of Helsinki [17]. The Northern Swedish Population Health Study (NSPHS) was approved by the local ethics committee at the University of Uppsala (Regionala Etikprövningsnämnden, Uppsala). The Scottish ORCADES study was approved by the NHS Orkney Research Ethics Committee and the North of Scotland REC. The Croatian VIS study was approved by the ethics committee of the medical faculty in Zagreb and the Multi-Centre Research Ethics Committee for Scotland. The Dutch ERF study was approved by the Erasmus institutional medical ethics committee in Rotterdam, The Netherlands. The Italian MICROS study was approved by the ethical committee of the Autonomous Province of Bolzano, Italy.

### Participants

The examined subjects stem from five different population-representative, pedigree-based cohorts from the EUROSPAN consortium (http://www.eurospan.org). All studies include a comprehensive collection of data on family structure, lifestyle, blood samples for clinical chemistry, RNA and DNA analyses, medical history, and current health status. All participants gave their written informed consent [18]. A brief description of each population is given below:

The *Northern Swedish Population Health Study* (NSPHS) represents a cross-sectional study conducted in the community of Karesuando in the subartic region of the County of Norrbotten, Sweden, in 2006 [5]. This parish has about 1500 eligible inhabitants of whom 740 participated in the study. The final sample consisted of 309 men and 347 women who were aged between 14 and 91 years. The inclusion of diet and activity covariates in the analytical model and according missing values reduced the effective sample size by less than 5%.

The *Orkney Complex Disease Study* (ORCADES) is a longitudinal study in the isolated Scottish archipelago of Orkney [19]. Participants from a subgroup of ten islands (*N* = 719) were used for the presented analysis. The sample comprised 334 men and 385 women aged between 18 and 100 years. The inclusion of diet and activity covariates in the analytical model and according missing values reduced the effective sample size by less than 5%.

The VIS study is a cross-sectional study in the villages of Vis and Komiza on the Dalmatian island of Vis, Croatia, and was conducted between 2003 and 2004 [20]–[22]. 795 participants who had both genotype and phenotypic data available were analysed. This cohort included 328 men and 467 women with an age between 18 and 93 years.

The *Microisolates in South Tyrol Study* (MICROS) is a cross-sectional study carried out in the villages of Stelvio, Vallelunga, and Martello, Venosta valley, South Tyrol, Italy, from 2001 to 2003 [23]. The 1,097 participants (475 males, 622 females, age between 18 and 88 years) presented in this study are those for whom both relevant genotype and phenotype data were available.

The *Erasmus Rucphen Family Study* (ERF) is a longitudinal study on a population living in the Rucphen region, the Netherlands, in the 19th century [24]. Fasting total cholesterol, HDL cholesterol and triglyceride levels were available. LDL cholesterol was estimated using the Friedewald formula [25]. The 918 individuals included in this study consisted of the first series of participants with 354 men and 564 women aged between 18 and 92 years.

### Genotyping

DNA samples were genotyped according to the manufacturer's instructions on Illumina Infinium HumanHap300v2 or HumanCNV370v1 SNP bead microarrays. Both arrays have 311,388 SNP markers in common that are distributed across the human genome. Analysis of the raw data was done in the BeadStudio software with the recommended parameters for the Infinium assay and using the genotype cluster files provided by Illumina. Individuals with a call rate below 95% and SNPs with a call rate below 98%, deviating from Hard-Weinberg equilibrium (*p*_HWE_<1×10^−6^) or with a minor allele frequency of less than 1% were excluded from the analysis.

### Lipids

Total cholesterol (TC), low-density lipoprotein cholesterol (LDL-C), high-density lipoprotein cholesterol (HDL-C), and triglycerides (TG) were quantified by enzymatic photometric assays using an ADVIA1650 clinical chemistry analyzer (Siemens Healthcare Diagnostics GmbH, Eschborn, Germany) at the Institute for Clinical Chemistry and Laboratory Medicine, Regensburg University Medical Center, Germany.

### Diet

In the NSPHS cohort, we collected data with a food frequency questionnaire based on the Northern Sweden 84-item Food Frequency Questionnaire (NoS-84-FFQ) [26]. We included in the questionnaire several items on foods specific for the lifestyle in this geographic region, in particular on game consumption (reindeer, moose). The answer options consisted of an 11-point format: 0 = “Never”, 1 = “less than 1 time per month”, 2 = “1 to 3 times per month”, 3 = “1 time per week”, 4 = “2 to 4 times per week”, 5 = “5 to 6 times per week”, 6 = “1 time per day”, 7 = “2 to 3 times per day”, 8 = “4 to 5 times per day”, 9 = “6 to 8 times per day”, 10 = “9 to 10 times per day”. The questionnaire was applied in electronic format by a trained study nurse as an interviewer. For each food item we calculated daily intake in gram per day as a standardized unit of measurement and aggregated the items to food categories, such game meat, non-game meat, fish, and dairy products. We evaluated the construct validity (known-groups validity) of the added items on game consumption in the NoS-84-FFQ questionnaire. We compared reindeer herders (*N* = 94) versus non-reindeer herders (*N* = 505). We observed highly significant, large effect sizes in men (*ES* = 1.25, *p* = 9.7×10^−04^) and women (*ES* = 1.15, p = 2.9×10^−05^) in the expected direction corresponding with an approximately three times higher consumption of absolute overall game intake in reindeer herders compared to others. A similar approach was used for the measurement and analysis of dietary data collected with a food frequency questionnaire in the Scottish cohort (Table S2).

### Physical activity

In the NSPHS cohort, we used two self-report scales to measure overall physical activity at work and at leisure. The Work Activity Scale (WAS, 6 items) addresses typical occupational physical activities: sitting, standing, walking, lifting, and general indicators of physical activity, i. e. sweating and tiredness after work. The Leisure Activity Scale (LAS, 4 items) asks for various typical freetime activities such walking, cycling, other sporting activities, and sweating as a general indicator of physical activity. Participants reported the frequency of each activity on a 5-point rating scale (1 = “never”, 2 = “seldom”, 3 = “sometimes”, 4 = “often”, and 5 = “always”). Both scales showed satisfying internal consistency with Cronbach's α(WAS) = 0.73 and Cronbach's α(LAS) = 0.70. A similar approach was used for the measurement and analysis of data on physical activity collected with a self-report questionnaire in the Scottish cohort (Table S2).

### Statistical analysis

#### Model selection

Sex and age are chosen as standard moderators of medical outcomes. Food and physical activity covariates have been selected based on findings on natural variation in lifestyle factors in this (data not presented) and other [7] northern Swedish populations between a modern, sedentary and a traditional, semi-nomadic lifestyle based on reindeer herding. Mostly significant associations between diet and activity covariates and lipid levels were found in the examined Swedish EUROSPAN cohort in the following ranges: *r* = [−0.01;0.12] (*p* = [1.28×10^−02^;0.16]) for game meat, *r* = [−0.13;−0.05] (*p* = [8.63×10^−04^;0.74]) for non-game meat, *r* = [0.06;0.16] (*p* = [2.12×10^−05^;0.12]) for fish, *r* = [0.04;0.13] (*p* = [2.51×10^−09^;3.85×10^−06^]) for physical activity at work, and *r* = [−0.11;0.01] (*p* = [5.05×10^−09^;1.30×10^−06^]) for physical activity at leisure (Table S3). We finally selected sex, age, game meat, non-game meat, fish, dairy products, physical activity at work, and physical activity at leisure as covariates in our diet- and activity-adjusted model (“adjusted” model) in the Swedish EUROSPAN sample. Sex and age were used as covariates in the “unadjusted” model.

We tested whether the inclusion of those covariates in the explanatory model led to a statistical significant improvement of the goodness of model fit compared to a restricted model by applying a maximum likelihood ratio (*MLR*) test. We inferred a significant better model fit of the full model if the difference of the χ^2^ value between both models had an equal or lower probability than *p* = 0.05 (one-sided, upper tail) on a χ^2^ distribution with *k* degrees of freedom. The degrees of freedom *k* are equal to the difference of the number of parameters in each model. The difference of χ^2^ values between both models is calculated according to the following formula with *MLE* indicating the maximum likelihood estimates per model: χ^2^(rest−full) = −2 (*log*_10_(*MLE*_rest_)−*log*_10_(*MLE*_full_)). The comparison of the goodness of fit between the unadjusted and the diet- and activity-adjusted full model, using a *MLR* test, showed a statistically significant improvement for all four lipid traits (TC: χ^2^_diff_ = 59.69, *df* = 6, *p* = 5.21×10^−11^; LDL-C: χ^2^_diff_ = 39.45, *df* = 6, *p* = 5.85×10^−07^; HDL-C: χ^2^_diff_ = 29.57, *df* = 6, *p* = 4.75×10^−05^; TG: χ^2^_diff_ = 69.32, *df* = 6, *p* = 5.65×10^−13^). All included polygenic, anthropometric and lifestyle factors (with the effect of including only the polygenic, sex, and age effects in parentheses) explained 64.07% (58.02%) of the variation of TC, 59.47% (56.47%) of the variation of LDL-C, 83.73% (82.59%) of the variance of HDL-C and 58.68% (41.80%) of the variation of TG levels. Dietary measures accounted for 22% (TC), 40% (LDL-C), 74% (HDL-C), and 7% (TG), respectively, of the variance explained by lifestyle factors with physical activity being responsible for the rest. GWAS results for models adjusted for sex, age, and diet only (Figures S1A, S1B, S1C, S1D) or physical activity only (Figures S2A, S2B, S2C, S2D) are presented in the supporting figures.

The confounding effect of treatment with statins on total cholesterol level and LDL cholesterol level was adjusted for by imputing untreated lipid concentrations of medicated individuals using the *npsubtreated()* function of the *R/GenABEL* package which implements the algorithm of Tobin et al. [27]. Additionally, we conducted the same analysis in subsamples which did not receive any lipid-lowering treatment and found overall converging, but somewhat weaker results for rs2000999 (*p*_SE,adj_ = 2.55×10^−04^; *p*_SC,adj_ = 2.07×10^−02^, *p*_NS,unadj_ = 5.93×10^−02^), rs1532624 (*p*_SE,adj_ = 2.26×10^−05^; *p*_SC,adj_ = 2.28×10^−09^, *p*_NS,unadj_ = 2.37×10^−19^), and rs5400 (*p*_SE,adj_ = 5.34×10^−06^; *p*_SC,adj_ = 2.23×10^−01^, *p*_NS,unadj_ = 8.04×10^−02^) (Table S4).

#### Genome-wide association analysis

First, deviations from normality for all quantitative traits (lipids, age, diet, and physical activity) were corrected by inverse-normal transformation without adjusting for covariates. Second, linear mixed effects models were fitted for the transformed outcomes (TC, LDL-C, HDL-C, TG) using the above mentioned covariates in the Swedish EUROSPAN sample and corresponding measures in the Scottish EUROSPAN sample (Table S2). The analysis was performed using the “polygenic” linear mixed effects model function *polygenic()* of the *R/GenABEL* package. Third, genome-wide association analysis was performed using a score test, a family-based association test [28], implemented in the *mmscore()* function of *R/GenABEL.* It uses the residuals and the variance-covariance matrix from the polygenic model and additional the SNP fixed effect coded under an additive model (0 = A/A, 1 = A/B, 2 = B/B). Fourth, genome-wide significance of a genetic loci was based on a local type I error of α = 0.05/311 388 SNPs = 1.6×10^−7^ according to a Bonferroni adjustment.

#### Candidate gene association analysis

The same statistical approach was used for association analysis of candidate loci with a local type I error of α = 0.05. No Bonferroni adjustment was applied to protect against α inflation since this method would be biased for the following reasons. The applied selection procedure for candidate loci makes the assumption of a global null hypothesis highly unlikely. Additionally, the phenotypes and some of the genotypes are highly correlated decreasing the number of independent tests. Instead all confirmatory tests are reported to allow the reader to evaluate the overall significance of the findings [29].

#### Relatedness

λ coefficients of lifestyle-adjusted genome-wide analysis varied in a low range between 1.00 and 1.04 in the Swedish cohort (see QQ-plots, Figures S3A, S3B, S3C, S3D, and Figure S4A, S4B, S4C, S4D) and between 1.00 and 1.01 in the Scottish cohort across all lipid traits. λ values for the unadjusted model used in the other three EUROSPAN cohorts did not exceed 1.01. These values indicate that our statistical model adequately handled relatedness in our pedigree-based samples since deflation of λ values is expected after correction for family structure.

#### Software and databases

We performed all analysis with the statistical analysis system *R* (V2.8.1) [30] mainly using the packages *GenABEL* (V1.4.2) [31] and *biomarRt* (V1.16.0) [32]. We accessed the following databases: *Ensembl* (http://www.ensembl.org) and *Online Mendelian Inheritance in Men* (http://www.ncbi.nlm.nih.gov/omim/).

## Supporting Information

Figure S1 Manhattan plots of genome-wide effects on total cholesterol, LDL cholesterol, HDL cholesterol, and triglyceride levels in the Swedish discovery cohort. Results for two GWAS analysis models are presented. The unadjusted model (dark blue and light blue circles) included only sex and age as covariates. The adjusted model (red and orange squares) additionally contained dietary measures (game meat, non-game meat, fish, milk products) as predictors. The dashed line indicates the local Bonferroni-adjusted α error = 1.6×10^−7^.
(0.31 MB DOC)

Figure S2 Manhattan plots of genome-wide effects on total cholesterol, LDL cholesterol, HDL cholesterol, and triglyceride levels in the Swedish discovery cohort. Results for two GWAS analysis models are presented. The unadjusted model (dark blue and light blue circles) included only sex and age as covariates. The adjusted model (red and orange squares) additionally contained physical activity measures (job, leisure) as predictors. The dashed line indicates the local Bonferroni-adjusted α error = 1.6×10^−7^.
(0.31 MB DOC)

Figure S3 QQ-Plots for the unadjusted GWAS on total cholesterol, LDL cholesterol, HDL cholesterol, and triglyceride levels in the Swedish discovery cohort. The analysis model was only adjusted for sex and age, but not for diet and activity measures (black line = expected slope under no inflation, red line = slope fitted to observations).
(0.12 MB DOC)

Figure S4 QQ-Plots for the adjusted GWAS on total cholesterol, LDL cholesterol, HDL cholesterol, and triglyceride levels in the Swedish discovery cohort. The analysis model was adjusted for sex, age, diet and activity measures (black line = expected slope under no inflation, red line = slope fitted to observations).
(0.12 MB DOC)

Table S1 GWAS results for all top candidate SNPs (0.05%) in the Swedish (SE) discovery cohort, the Scottish (SC), and all non-Swedish (NS) replication cohorts.
(0.41 MB XLS)

Table S2 Comparison of the diet- and activity-adjusted analysis model in the Swedish and the Scottish cohort.
(0.04 MB DOC)

Table S3 Pearson correlations, determination coefficients (explained variance), and *p*-values of the inverse-normal transformed lipid, dietary, and physical activity measures in the Swedish cohort.
(0.03 MB XLS)

Table S4 GWAS results for all top SNPs (0.05%) in the Swedish (SE) discovery cohort, and for all candidate SNPs in the Scottish (SC), and in the non-Swedish (NS) replication cohorts including only individuals without lipid-lowering treatment.
(0.34 MB XLS)
