# Supplementary material for: Modeling of Environmental Effects in Genome-Wide Association Studies Identifies SLC2A2 and HP as Novel Loci Influencing Serum Cholesterol Levels
Source: PLoS Genet. 2010 Jan 8;6(1):e1000798. doi: 10.1371/journal.pgen.1000798 (PMC2792712; doi:10.1371/journal.pgen.1000798)
Supplement: Figure S1 — Manhattan plots of genome-wide effects on total cholesterol, LDL cholesterol, HDL cholesterol, and triglyceride levels in the Swedish discovery cohort. Results for two GWAS analysis models are presented. The unadjusted model (dark blue and light blue circles) included only sex and age as covariates. The adjusted model (red and orange squares) additionally contained dietary measures (game meat, non-game meat, fish, milk products) as predictors. The dashed line indicates the local Bonferroni-adjusted α error = 1.6×10^−7^. (0.31 MB DOC) [file pgen.1000798.s001.doc]

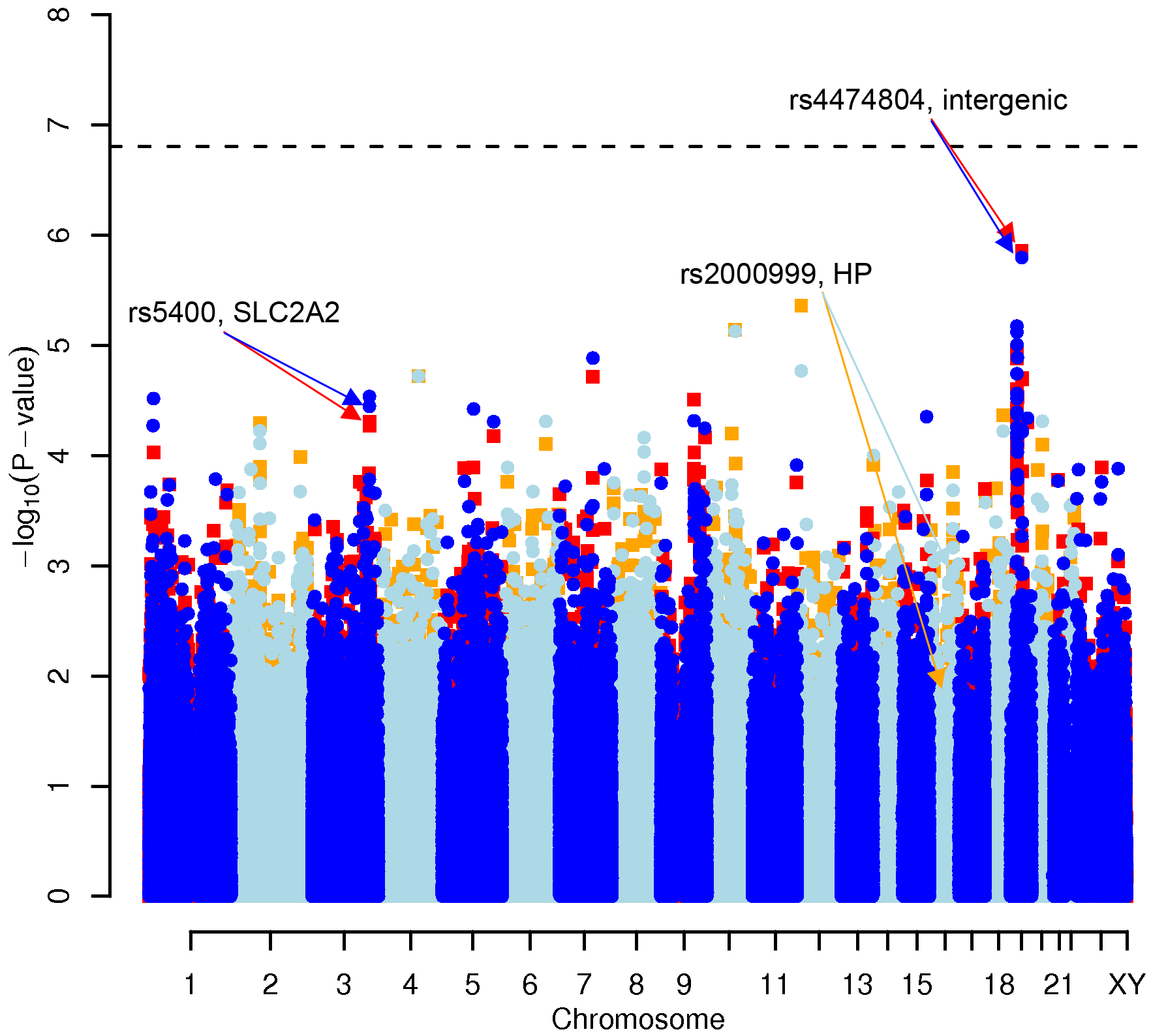
**Figure S1a. Manhattan plot of genome-wide effects on total cholesterol levels in the Swedish discovery cohort.** Results for two GWAS analysis models are presented. The unadjusted model (dark blue and light blue circles) included only sex and age as covariates. The adjusted model (red and orange squares) additionally contained dietary measures (game meat, non-game meat, fish, and milk products) as predictors. The dashed line indicates the local Bonferroni-adjusted α error = 1.6×10^-7^.


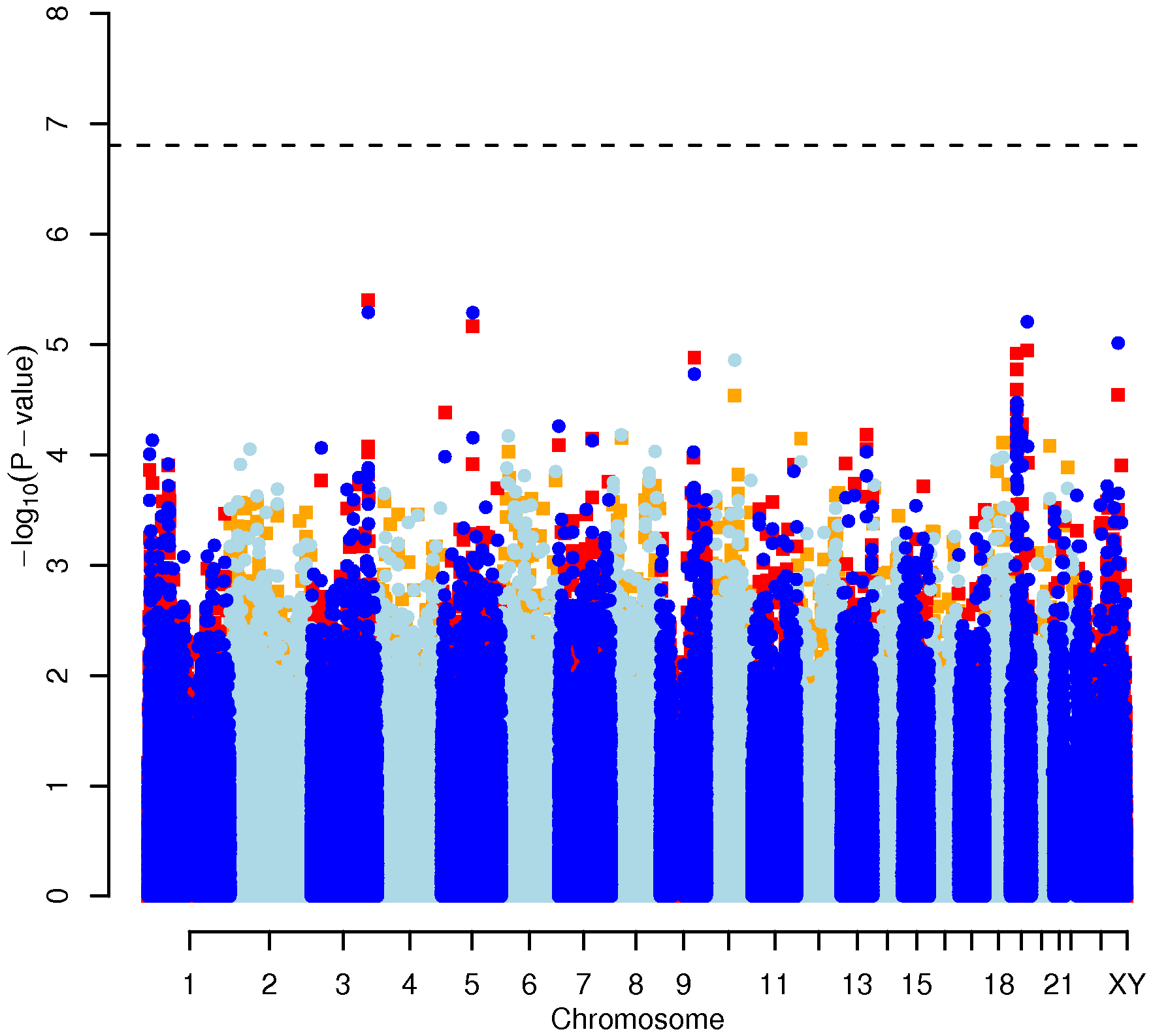
**Figure S1b. Manhattan plot of genome-wide effects on LDL cholesterol levels in the Swedish discovery cohort.** Results for two GWAS analysis models are presented. The unadjusted model (dark blue and light blue circles) included only sex and age as covariates. The adjusted model (red and orange squares) additionally contained dietary measures (game meat, non-game meat, fish, and milk products) as predictors. The dashed line indicates the local Bonferroni-adjusted α error = 1.6×10^-7^.


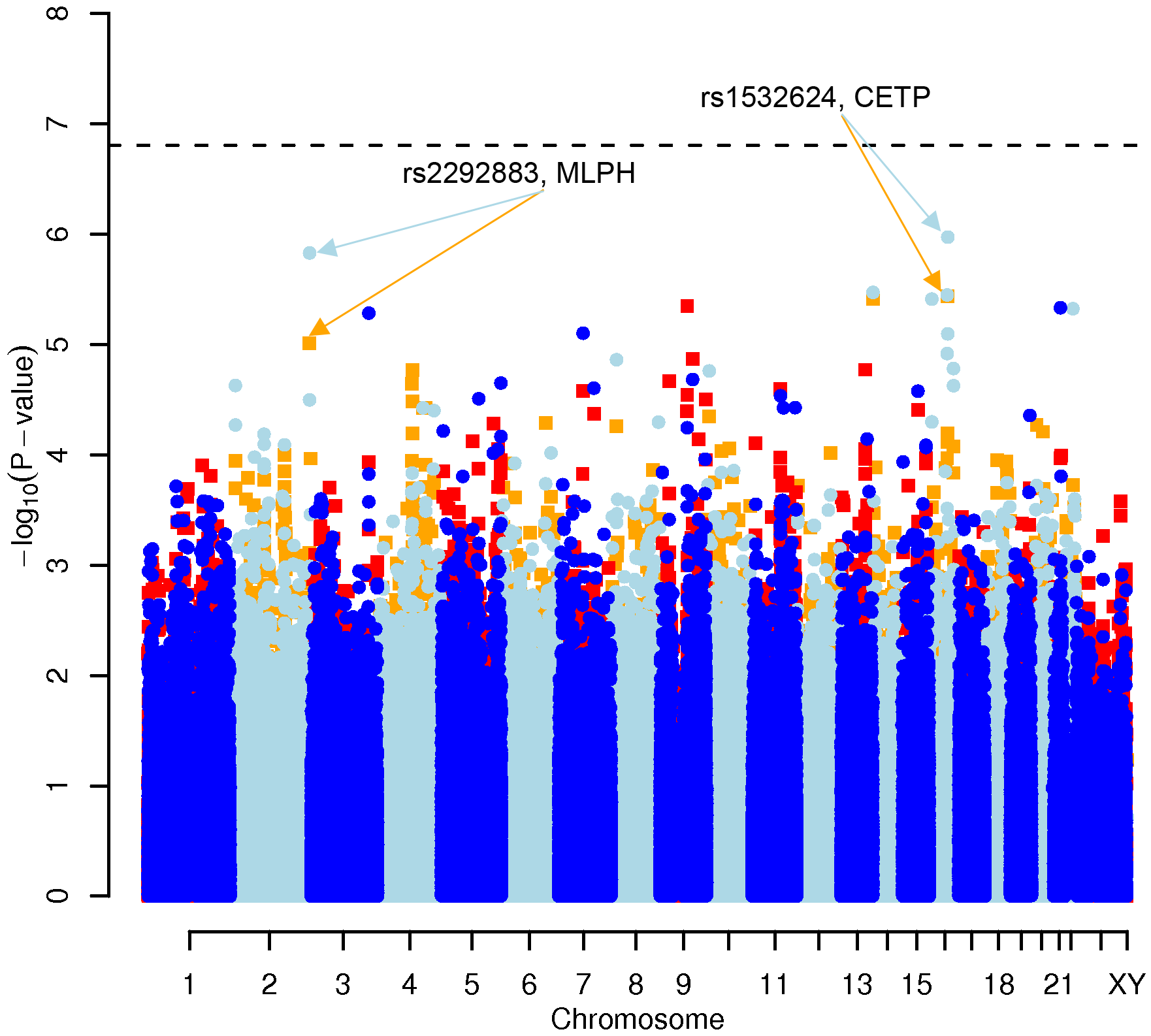
**Figure S1c. Manhattan plot of genome-wide effects on HDL cholesterol levels in the Swedish discovery cohort.** Results for two GWAS analysis models are presented. The unadjusted model (dark blue and light blue circles) included only sex and age as covariates. The adjusted model (red and orange squares) additionally contained dietary measures (game meat, non-game meat, fish, and milk products) as predictors. The dashed line indicates the local Bonferroni-adjusted α error = 1.6×10^-7^.


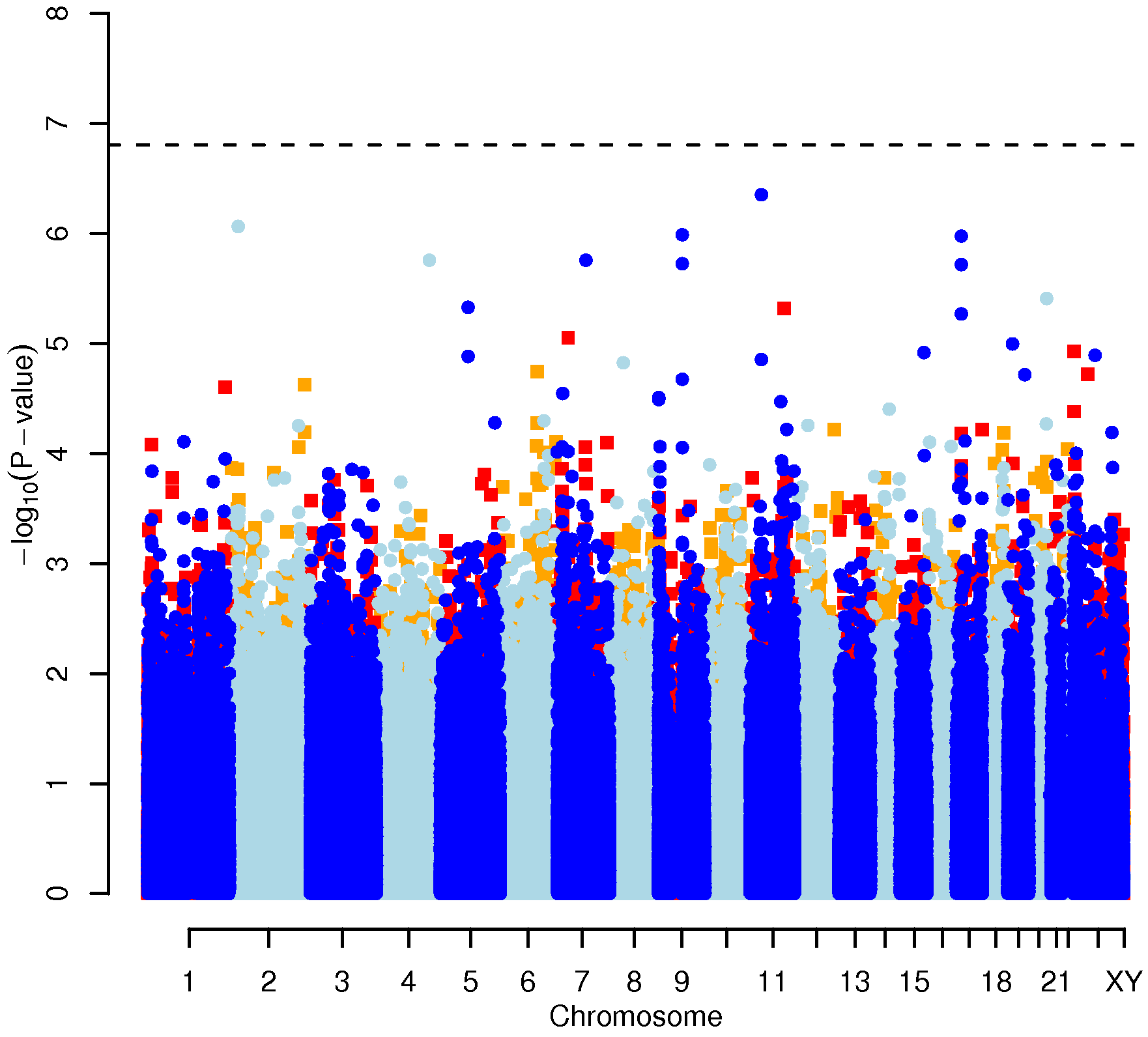
**Figure S1d. Manhattan plot of genome-wide effects on triglyceride levels in the Swedish discovery cohort.** Results for two GWAS analysis models are presented. The unadjusted model (dark blue and light blue circles) included only sex and age as covariates. The adjusted model (red and orange squares) additionally contained dietary measures (game meat, non-game meat, fish, and milk products) as predictors. The dashed line indicates the local Bonferroni-adjusted α error = 1.6×10^-7^.
