# Supplementary material for: Modeling of Environmental Effects in Genome-Wide Association Studies Identifies SLC2A2 and HP as Novel Loci Influencing Serum Cholesterol Levels
Source: PLoS Genet. 2010 Jan 8;6(1):e1000798. doi: 10.1371/journal.pgen.1000798 (PMC2792712; doi:10.1371/journal.pgen.1000798)
Supplement: Figure S2 — Manhattan plots of genome-wide effects on total cholesterol, LDL cholesterol, HDL cholesterol, and triglyceride levels in the Swedish discovery cohort. Results for two GWAS analysis models are presented. The unadjusted model (dark blue and light blue circles) included only sex and age as covariates. The adjusted model (red and orange squares) additionally contained physical activity measures (job, leisure) as predictors. The dashed line indicates the local Bonferroni-adjusted α error = 1.6×10^−7^. (0.31 MB DOC) [file pgen.1000798.s002.doc]

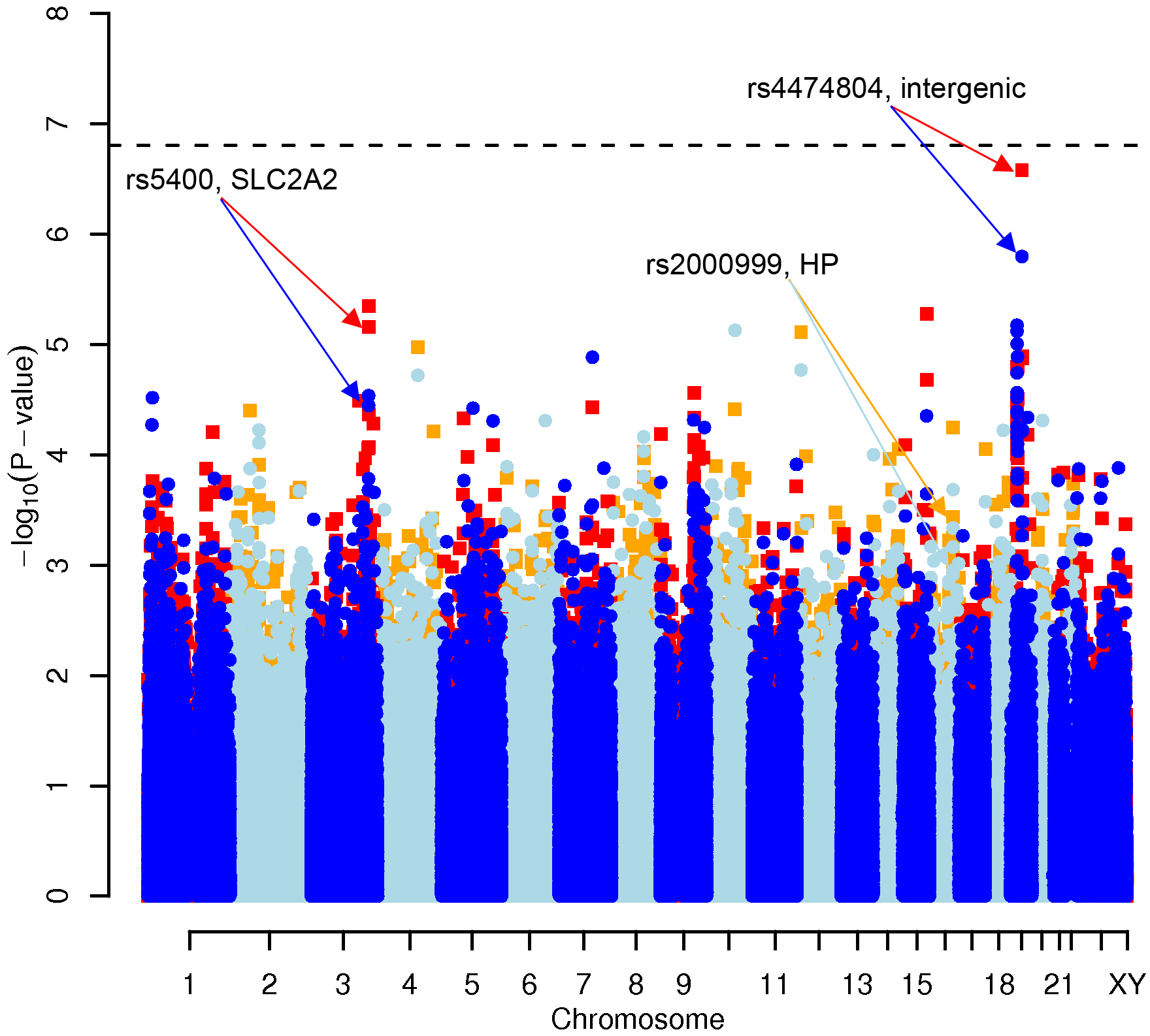
**Figure S2a. Manhattan plot of genome-wide effects on total cholesterol levels in the Swedish discovery cohort.** Results for two GWAS analysis models are presented. The unadjusted model (dark blue and light blue circles) included only sex and age as covariates. The adjusted model (red and orange squares) additionally contained physical activity measures (job, leisure) as predictors. The dashed line indicates the local Bonferroni-adjusted α error = 1.6×10^-7^.


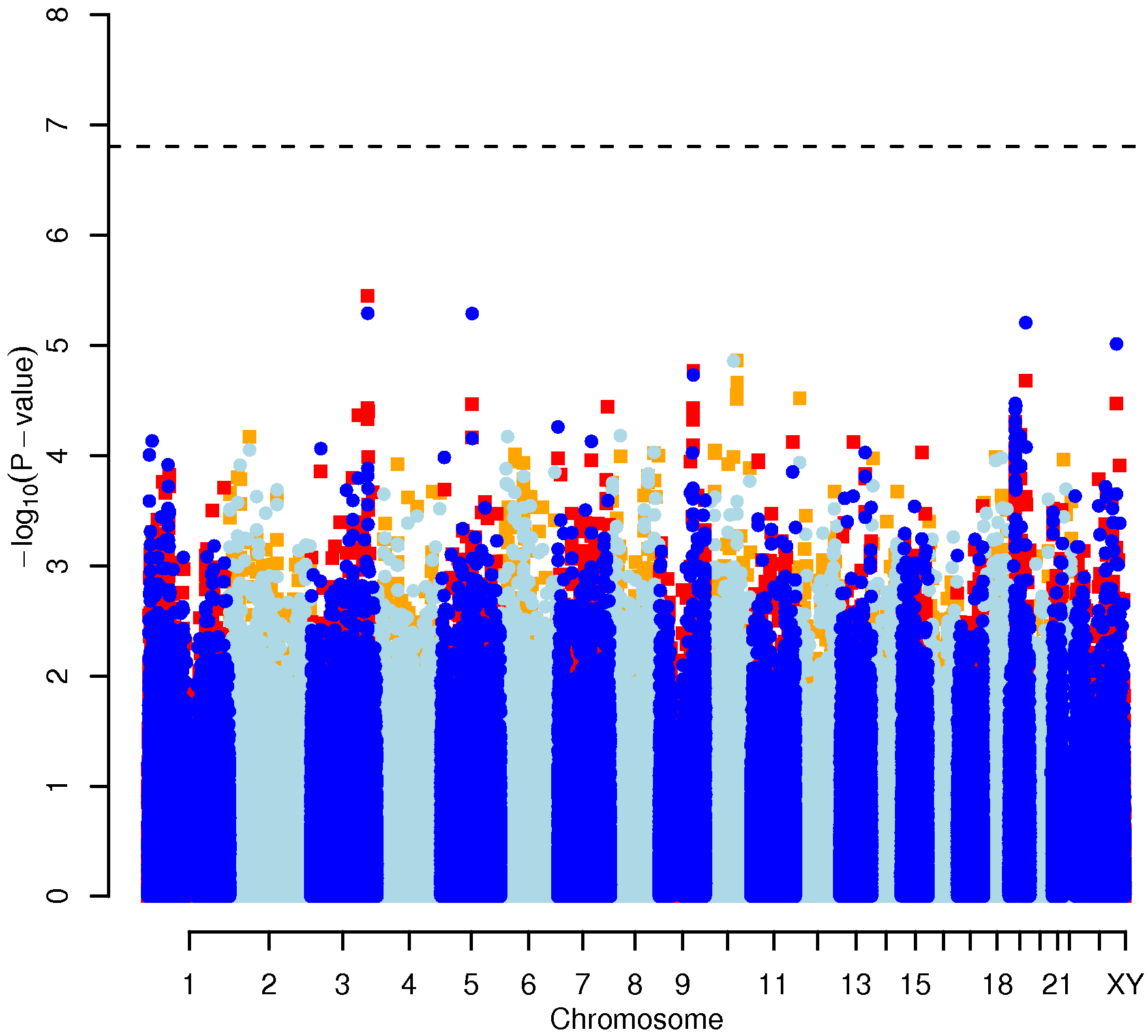
**Figure S2b. Manhattan plot of genome-wide effects on LDL cholesterol levels in the Swedish discovery cohort.** Results for two GWAS analysis models are presented. The unadjusted model (dark blue and light blue circles) included only sex and age as covariates. The adjusted model (red and orange squares) additionally contained physical activity measures (job, leisure) as predictors. The dashed line indicates the local Bonferroni-adjusted α error = 1.6×10^-7^.


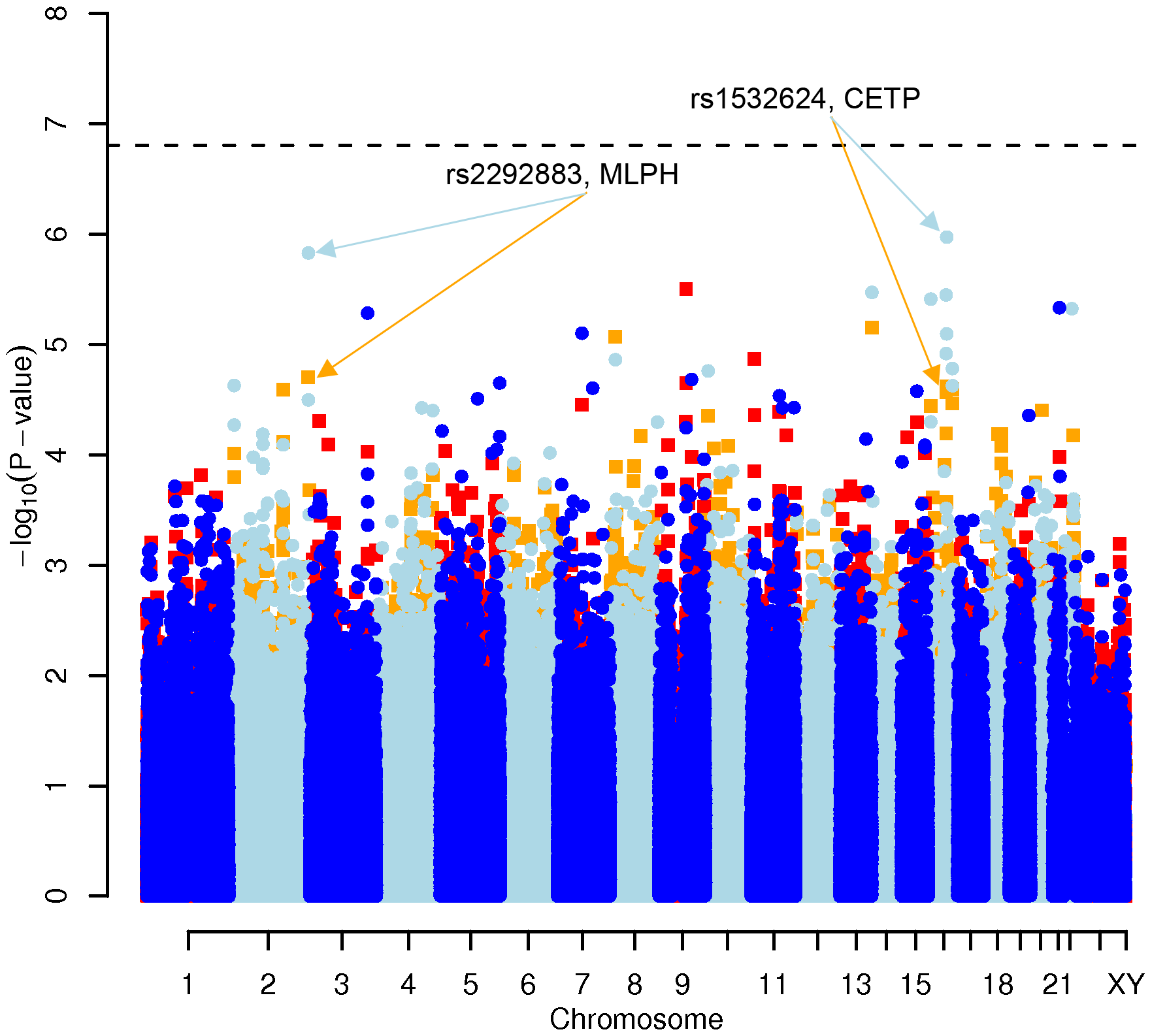
**Figure S2c. Manhattan plot of genome-wide effects on HDL cholesterol levels in the Swedish discovery cohort.** Results for two GWAS analysis models are presented. The unadjusted model (dark blue and light blue circles) included only sex and age as covariates. The adjusted model (red and orange squares) additionally contained physical activity measures (job, leisure) as predictors. The dashed line indicates the local Bonferroni-adjusted α error = 1.6×10^-7^.


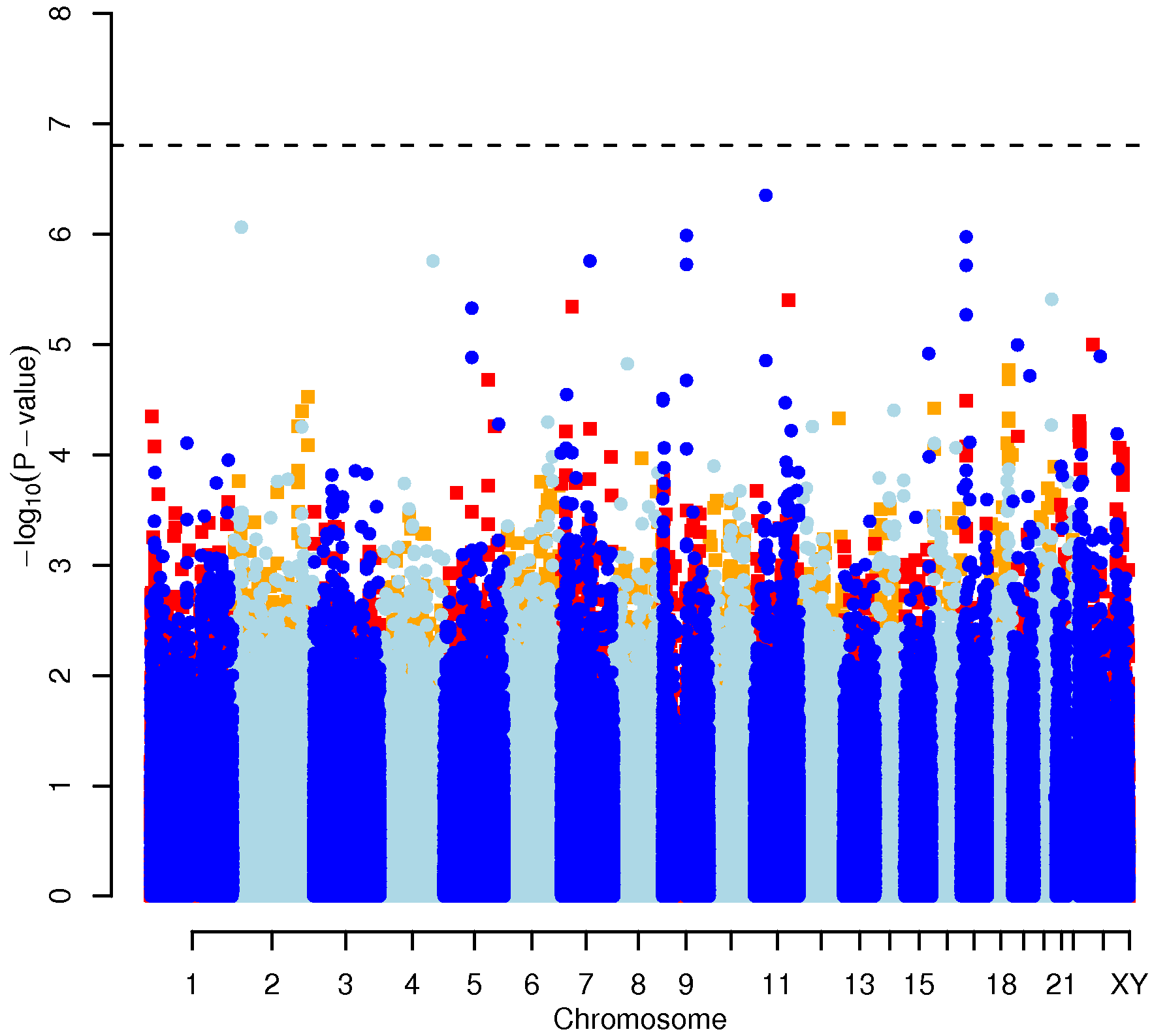
**Figure S2d. Manhattan plot of genome-wide effects on triglyceride levels in the Swedish discovery cohort.** Results for two GWAS analysis models are presented. The unadjusted model (dark blue and light blue circles) included only sex and age as covariates. The adjusted model (red and orange squares) additionally contained physical activity measures (job, leisure) as predictors. The dashed line indicates the local Bonferroni-adjusted α error = 1.6×10^-7^.
