# Supplementary material for: Modeling of Environmental Effects in Genome-Wide Association Studies Identifies SLC2A2 and HP as Novel Loci Influencing Serum Cholesterol Levels
Source: PLoS Genet. 2010 Jan 8;6(1):e1000798. doi: 10.1371/journal.pgen.1000798 (PMC2792712; doi:10.1371/journal.pgen.1000798)
Supplement: Figure S3 — QQ-Plots for the unadjusted GWAS on total cholesterol, LDL cholesterol, HDL cholesterol, and triglyceride levels in the Swedish discovery cohort. The analysis model was only adjusted for sex and age, but not for diet and activity measures (black line = expected slope under no inflation, red line = slope fitted to observations). (0.12 MB DOC) [file pgen.1000798.s003.doc]

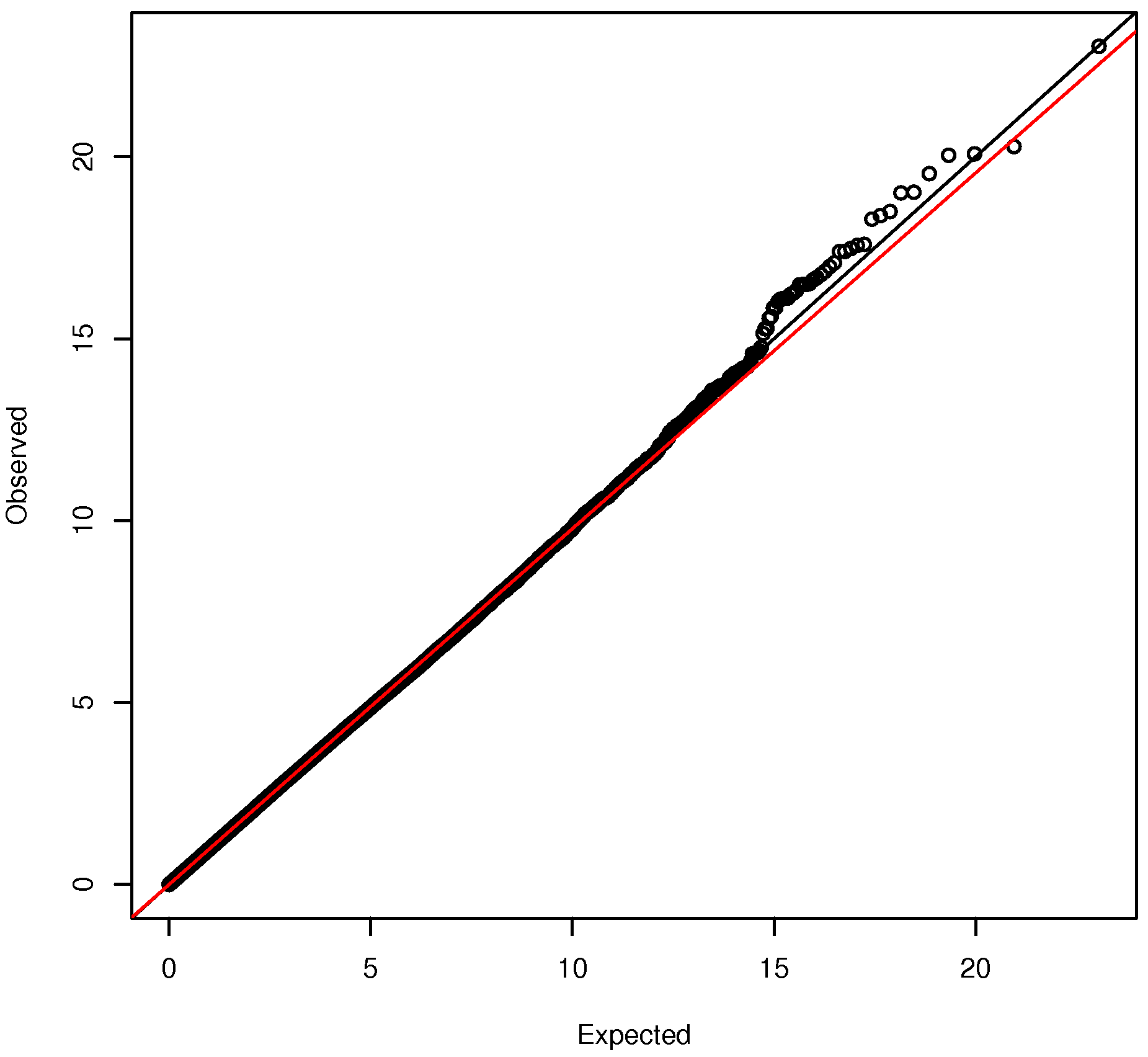
**Figure S3a. QQ-Plot for GWAS on total cholesterol level in the Swedish discovery cohort.** The analysis model was only adjusted for sex and age, but not for diet and activity measures (black line=expected slope under no inflation, red line= slope fitted to observations).


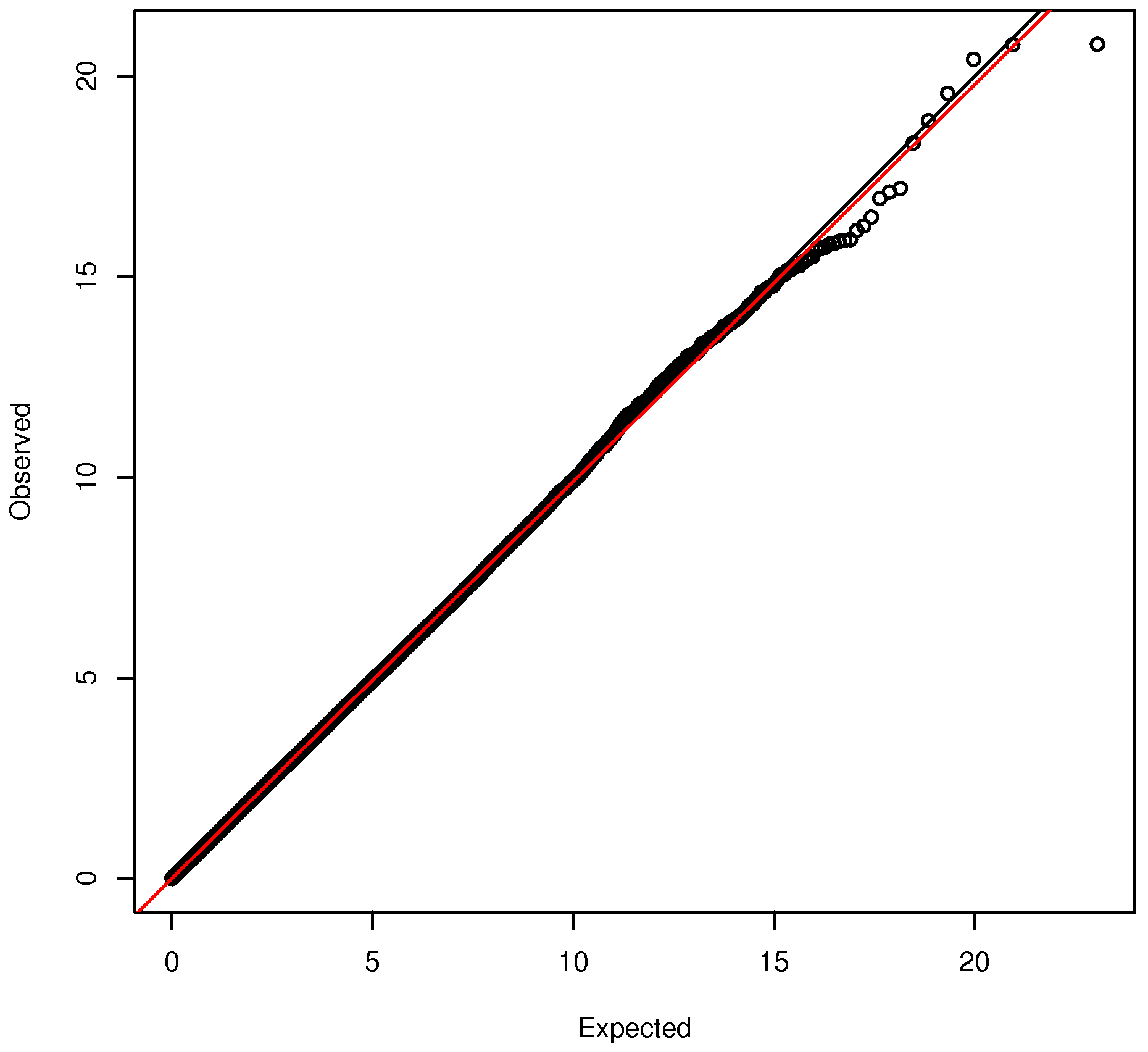
**Figure S3b. QQ-Plot for GWAS on LDL cholesterol level in the Swedish discovery cohort.** The analysis model was only adjusted for sex and age, but not for diet and activity measures (black line=expected slope under no inflation, red line= slope fitted to observations).


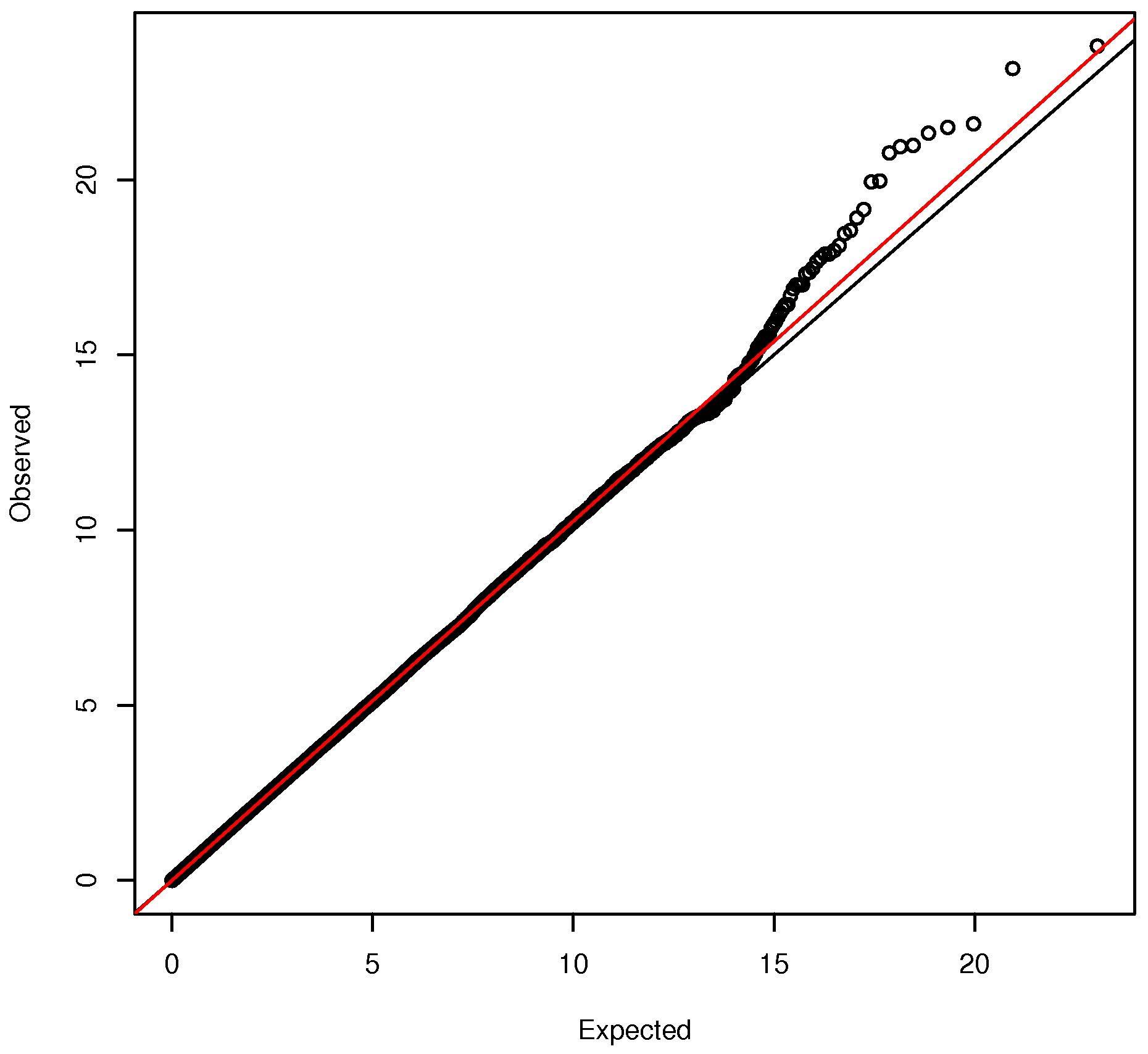
**Figure S3c. QQ-Plot for GWAS on HDL cholesterol level in the Swedish discovery cohort.** The analysis model was only adjusted for sex and age, but not for diet and activity measures (black line=expected slope under no inflation, red line= slope fitted to observations).


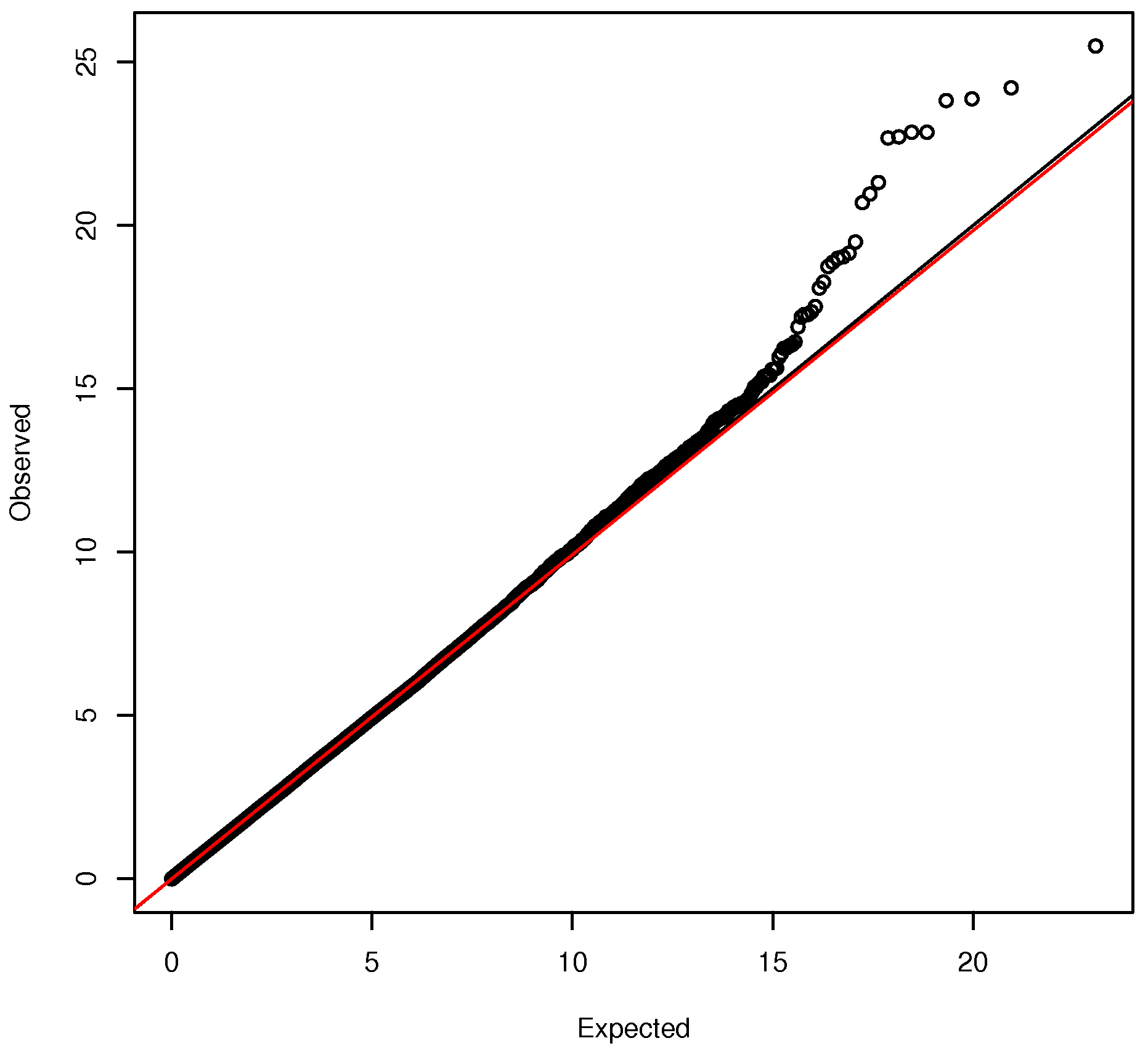
**Figure S3d. QQ-Plot for GWAS on triglyceride level in the Swedish discovery cohort.** The analysis model was only adjusted for sex and age, but not for diet and activity measures (black line=expected slope under no inflation, red line= slope fitted to observations).
