# Supplementary material for: Modeling of Environmental Effects in Genome-Wide Association Studies Identifies SLC2A2 and HP as Novel Loci Influencing Serum Cholesterol Levels
Source: PLoS Genet. 2010 Jan 8;6(1):e1000798. doi: 10.1371/journal.pgen.1000798 (PMC2792712; doi:10.1371/journal.pgen.1000798)
Supplement: Figure S4 — QQ-Plots for the adjusted GWAS on total cholesterol, LDL cholesterol, HDL cholesterol, and triglyceride levels in the Swedish discovery cohort. The analysis model was adjusted for sex, age, diet and activity measures (black line = expected slope under no inflation, red line = slope fitted to observations). (0.12 MB DOC) [file pgen.1000798.s004.doc]

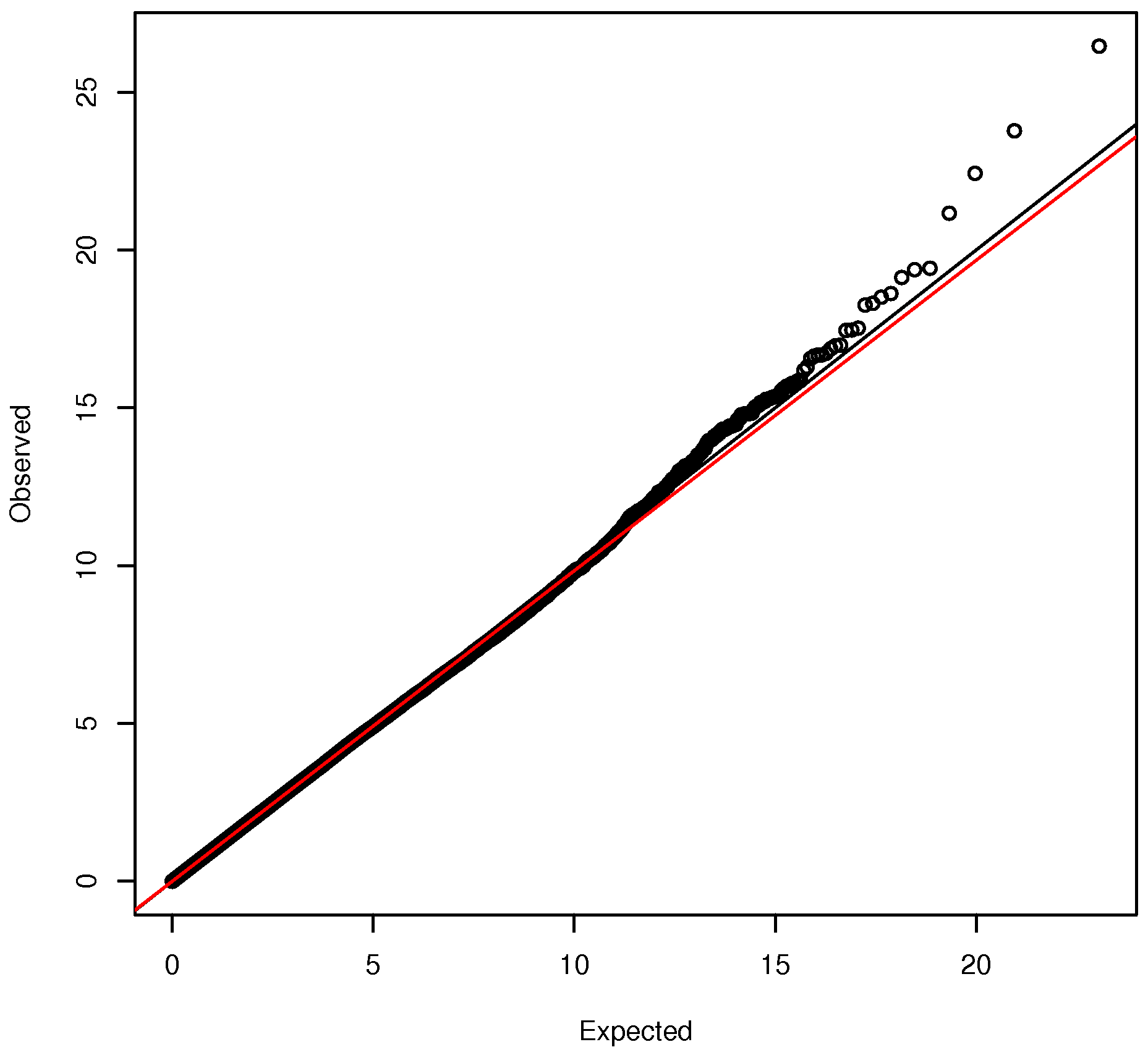
**Figure S4a. QQ-Plot for GWAS on total cholesterol level in the Swedish discovery cohort.** The analysis model was adjusted for sex, age, diet and activity measures (black line=expected slope under no inflation, red line= slope fitted to observations).


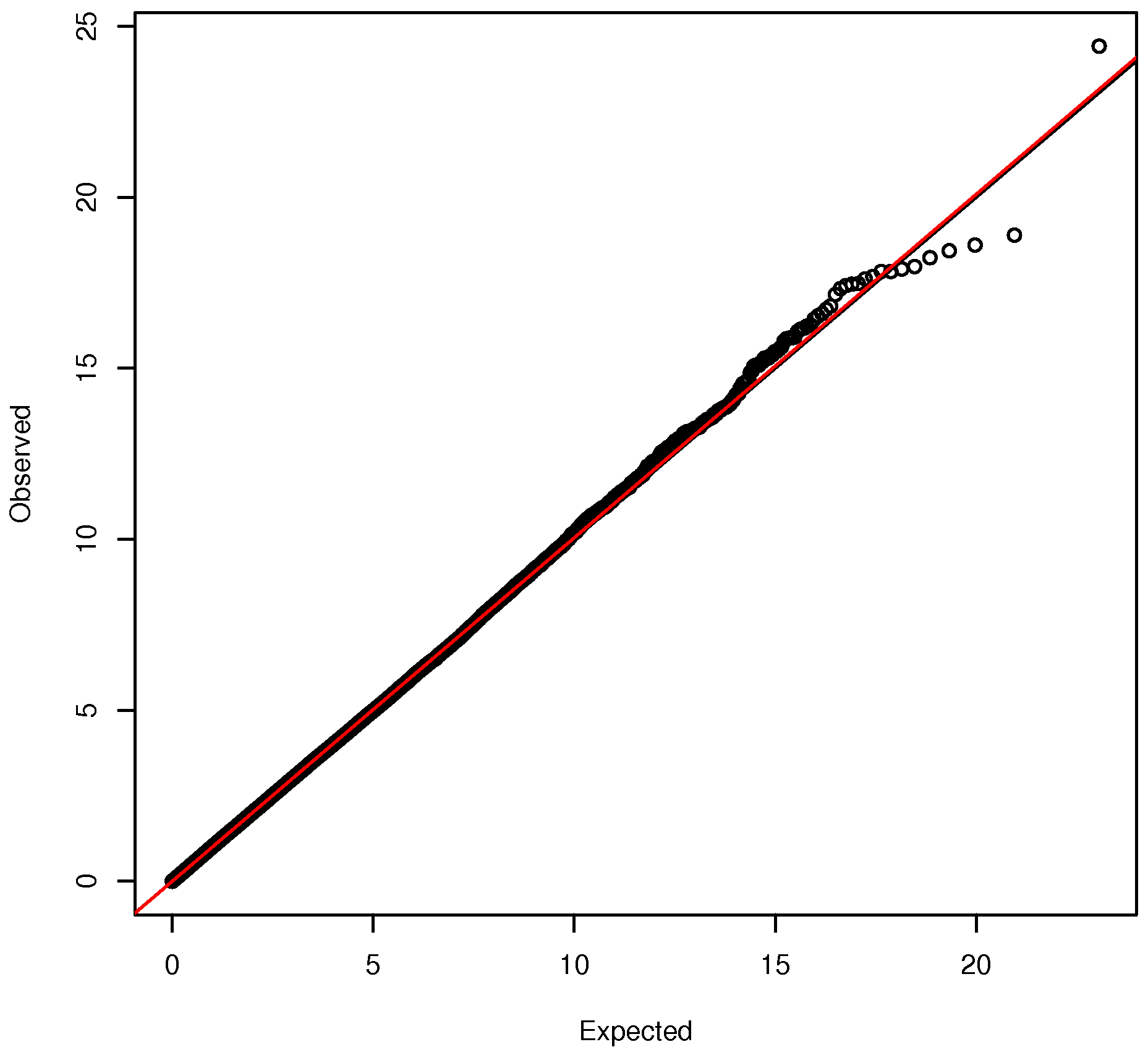
**Figure S4b. QQ-Plot for GWAS on LDL cholesterol level in the Swedish discovery cohort.** The analysis model was adjusted for sex, age, diet and activity measures (black line=expected slope under no inflation, red line= slope fitted to observations).


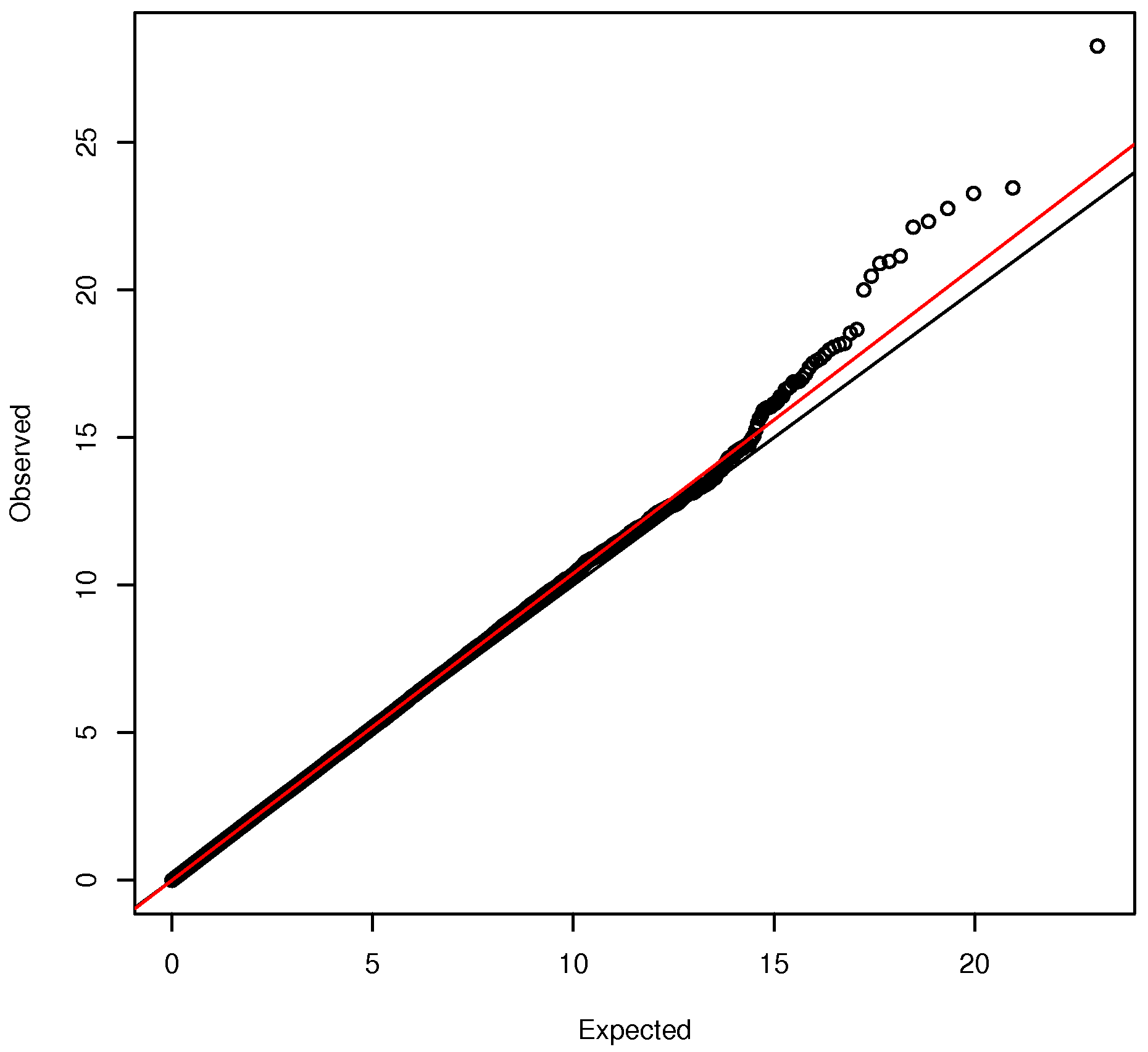
**Figure S4c. QQ-Plot for GWAS on HDL cholesterol level in the Swedish discovery cohort.** The analysis model was adjusted for sex, age, diet and activity measures (black line=expected slope under no inflation, red line= slope fitted to observations).


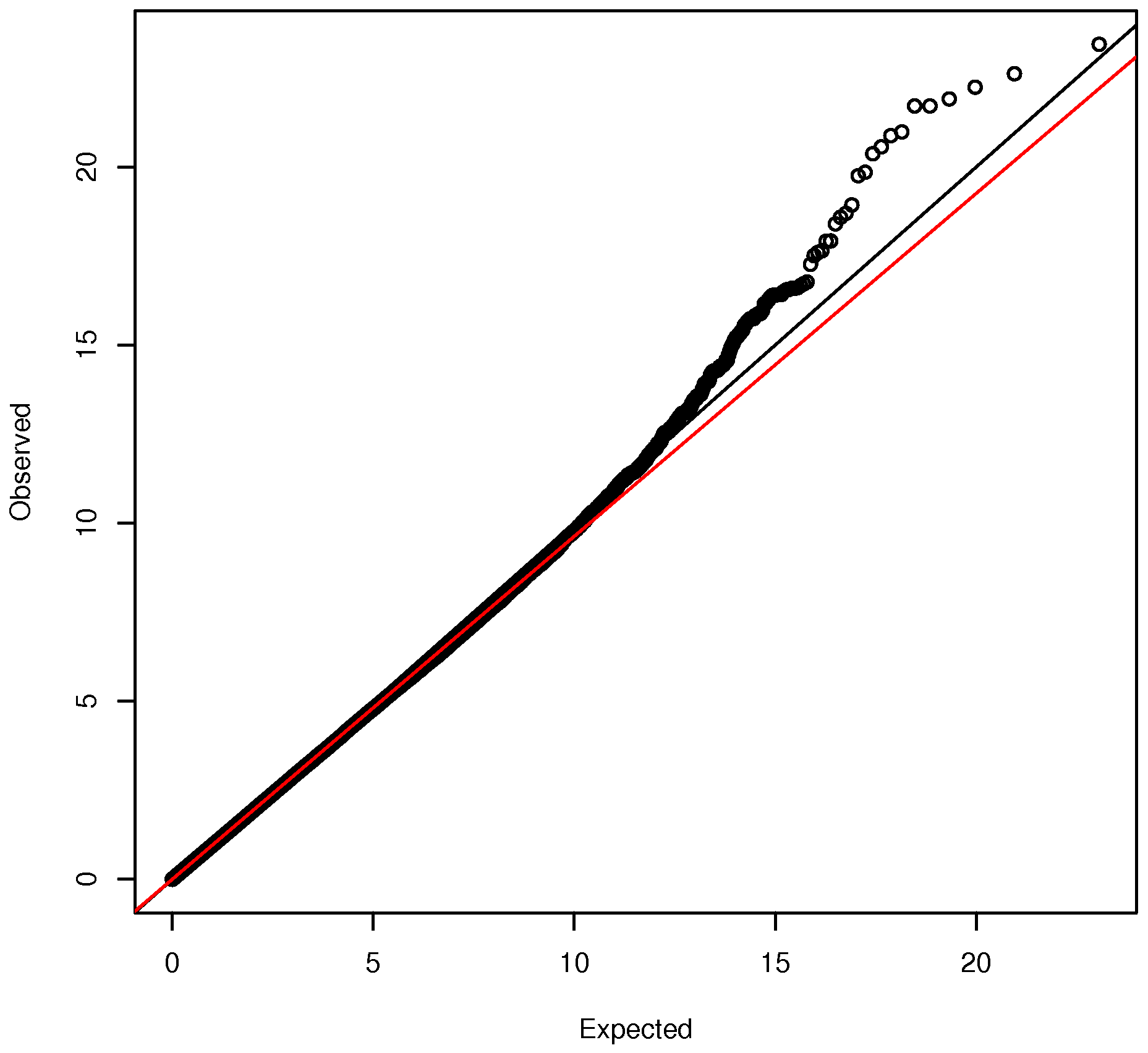
**Figure S4d. QQ-Plot for GWAS on triglyceride level in the Swedish discovery cohort.** The analysis model was adjusted for sex, age, diet and activity measures (black line=expected slope under no inflation, red line= slope fitted to observations).
