# Supplementary material for: Modeling of Environmental Effects in Genome-Wide Association Studies Identifies SLC2A2 and HP as Novel Loci Influencing Serum Cholesterol Levels
Source: PLoS Genet. 2010 Jan 8;6(1):e1000798. doi: 10.1371/journal.pgen.1000798 (PMC2792712; doi:10.1371/journal.pgen.1000798)
Supplement: Table S2 — Comparison of the diet- and activity-adjusted analysis model in the Swedish and the Scottish cohort. (0.04 MB DOC) [file pgen.1000798.s006.doc]

**Table S2. Comparison of the diet- and activity-adjusted analysis model in the Swedish and the Scottish cohort**.

| **Construct** | **Model** | **Transformation** | **Variable Label** | **Value Label/Measurement Unit (Swedish Cohort, NSPHS)** | **Value Label/Measurement Unit (Scottish Cohort, ORCADES)** |
| --- | --- | --- | --- | --- | --- |
| **Criteria** |  |  |  |  |  |
| Lipid | Criterion | log-normal | Total Cholesterol (TC) | mg/dl | mg/dl |
| Lipid | Criterion | log-normal | Low-density lipoprotein cholesterol (LDL-C) | mg/dl | mg/dl |
| Lipid | Criterion | log-normal | High-density lipoprotein cholesterol (HDL-C) | mg/dl | mg/dl |
| Lipid | Criterion | log-normal | Triglycerides (TG) | mg/dl | mg/dl |
| **Covariates** |  |  |  |  |  |
| Sociodemography | Covariate, fixed | untransformed | Sex | 0=female, 1=male | 0=female, 1=male |
| Sociodemography | Covariate, fixed | log-normal | Age | Years | Years |
| Diet | Covariate, fixed | log-normal | Meat (non-game or “red” meat) | Frequency of intake of non-game meat (0="Never", 1="Less than 1 time per month", 2="1 to 3 times per month", 3="1 time per week", 4="2 to 4 times per week", 5="5 to 6 times per week", 6="1 time per day", 7="2 to 3 times per day", 8="4 to 5 times per day", 9="6 to 8 times per day", 10="9 to 10 times per day", converted to gram per day) | Frequency of intake of “red” meat (0=”Less often or never”, 1=”1-3 times a month”, 2=”Once a week”, 3=”2-4 times a week”, 4=”5-6 times a week”, 5=”1 portion a day”, 6=”2-3 portions a day”, 7=”4-5 portions a day”, 8=”6 or more portions a day”, converted to gram per day) |
| Diet | Covariate, fixed | log-normal | Meat (game or “white” meat) | Frequency of intake of game meat (cf. above , converted to gram per day) | Frequency of intake of “white” meat (cf. above, converted to gram per day) |
| Diet | Covariate, fixed | log-normal | Fish | Frequency of fish intake (cf. above, converted to gram per day) | Frequency of fish intake (cf. above, converted to gram per day) |
| Diet | Covariate, fixed | log-normal | Dairy Products | Frequency of dairy product intake (cf. above, converted to gram per day) | Frequency of dairy product intake (cf. above, converted to gram per day) |
| Physical Activity | Covariate, fixed | log-normal | Physical Activity, Leisure | 1="Never", 2="Seldom", 3="Sometimes", 4="Often", 5="Very often" | 1=”Light”, 2=”Moderate” |
| Physical Activity | Covariate, fixed | log-normal | Physical Activity, Work | 1="Never", 2="Seldom", 3="Sometimes", 4="Often", 5="Very often" | 1=”Mostly sitting down”, 2=”Mostly standing up”, 3=”Manual work”, 4=”Heavy manual work” |
| Genotype | Covariate, random | untransformed | Kinship cluster | categorical | categorical |
| Genotype | Predictor, fixed | untransformed | Single-Nucleotide Polymorphism | 0=AA,1=AB,2=BB (additive model) | 0=AA,1=AB,2=BB (additive model) |
